# Stability of fruit quality traits in diverse watermelon cultivars tested in multiple environments

**DOI:** 10.1038/hortres.2016.66

**Published:** 2016-12-21

**Authors:** Mahendra Dia, Todd C Wehner, Penelope Perkins-Veazie, Richard Hassell, Daniel S Price, George E Boyhan, Stephen M Olson, Stephen R King, Angela R Davis, Gregory E Tolla, Jerome Bernier, Benito Juarez

**Affiliations:** 1 Department of Horticultural Science, North Carolina State University, Raleigh, NC 27695-7609, USA; 2 Plants for Human Health Institute, North Carolina Research Campus NCSU, Kannapolis, NC 28081, USA; 3 Clemson University, Coastal Research and Education Center, Charleston, SC 29414, USA; 4 Georgia County Extension, SW District, Cordele, GA 31015, USA; 5 Department of Horticulture, University of Georgia, Athens, GA 30602, USA; 6 North Florida REC, University of Florida, Quincy, FL 32351-5677, USA; 7 Texas A&M University, Department of Horticulture Science, College Station, TX 77845, USA; 8 USDA-ARS, Lane, OK 74555, USA; 9 Monsanto/Seminis Veg. Seeds, Woodland, CA 95695, USA; 10 Sakata Seed America, Inc., 1833 Dunlap Pl., Woodland, CA 95776, USA

## Abstract

Lycopene is a naturally occurring red carotenoid compound that is found in watermelon. Lycopene has antioxidant properties. Lycopene content, sugar content and hollowheart resistance are subject to significant genotype×environment interaction (G×E), which makes breeding for these fruit quality traits difficult. The objectives of this study were to (i) evaluate the influence of years and locations on lycopene content, sugar content and hollowheart resistance for a set of watermelon genotypes, and (ii) identify genotypes with high stability for lycopene, sugar, and hollowheart resistance. A diverse set of 40 genotypes was tested over 3 years and 8 locations across the southern United States in replicated, multi-harvest trials. Lycopene was tested in a subset of 10 genotypes. Data were analyzed using univariate and multivariate stability statistics (BLUP-GGE biplot) using SASGxE and RGxE programs. There were strong effects of environment as well as G×E interaction on watermelon quality traits. On the basis of stability measures, genotypes were classified as stable or unstable for each quality trait. 'Crimson Sweet' is an inbred line with high quality trait performance as well as trait stability. 'Stone Mountain', 'Tom Watson', 'Crimson Sweet' and 'Minilee' were among the best genotypes for lycopene content, sugar content and hollowheart resistance. We developed a stability chart based on marketable yield and average ranking generated from different stability measures for yield attributes and quality traits. The chart will assist in choosing parents for improvement of watermelon cultivars. See http://cuke.hort.ncsu.edu/cucurbit/wmelon/wmelonmain.html.

## Introduction

Lycopene is a red carotenoid with antioxidant properties that provides human health benefits including protection against stroke and cardiovascular diseases, and reduced cancer cell growth. In red fleshed watermelon, multiple genes controlling carotenoid synthesis have been identified.^1^ They concluded that lycopene content in watermelon is inherited as a quantitative trait. Other fruit quality traits that are quantitatively inherited include hollowheart resistance and sugar content (measured as total soluble solids in °Brix).

**Figure 1:**
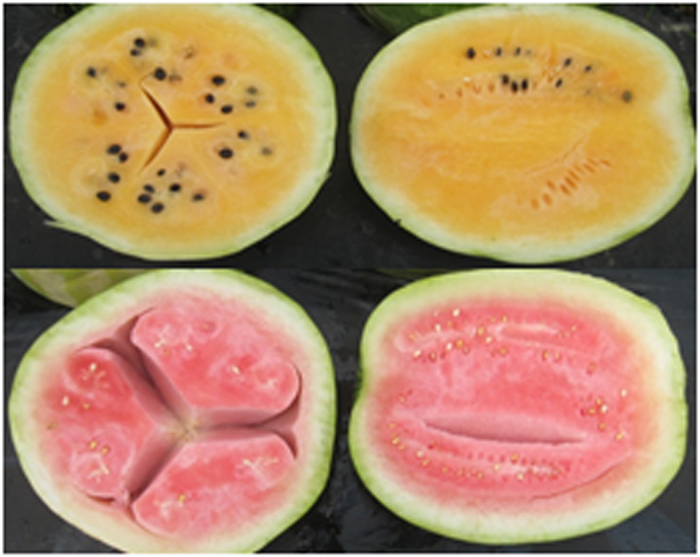
Hollowheart defect in watermelon.

Watermelon growers and shippers are interested in reducing the incidence of hollowheart. Hollowheart is characterized by the separation of flesh inside of the fruit (Figure 1). It is difficult to identify fruit that have hollowheart without cutting them. Little research has been done on its causes but it is affected by environment. Lou and Wehner^2^ studied the inheritance of hollowheart in two families of watermelon and found that it was non-Mendelian or quantitative. Similarly, the total soluble solids or sugar content is a major component of watermelon flavor. Sugar content in watermelon is polygenic.^3^ In similar studies, Jackson^4^ and Audilakshmi *et al.*^5^ reported polygenic inheritance of sugar content in sugarcane (*Saccharum officinarum*) and sweet sorghum (*Sorghum vulgar var. Saccaratum Mloench*).

Quantitative traits are often affected by environment, and a single gene can have different effects on trait performance in different regions. There are few examples of quantitative traits that were unaffected by environment. Genotype×environment interaction (G×E) occurs when there is a scale shift or a rank shift in genotype performance across environments. Hereafter, the word ‘genotype’ will be used to indicate cultigen, cultivar, variety or genotype. The presence of G×E makes it useful to measure both performance and stability of genotypes in a breeding program.^6^ G×E may result in a low correlation between phenotypic and genotypic values, thereby reducing progress from selection. This leads to bias in the estimation of heritability and in the prediction of genetic advance.^7,8^ Therefore, presence of G×E will change how selection and testing are done in a breeding program.

Several statistical methods for evaluating stability have been proposed. These include univariate models, such as regression slope, deviation from regression, and environmental variance; and multivariate models, such as genotype main effect plus genotype by environment interaction (GGE) biplot.^9–12^ No single method adequately explains genotype performance across environments. Our approach is to use stability statistics (variance) in combination with trait performance (mean).

Analysis of variance (ANOVA) is often used to identify the magnitude and statistical significance of G×E in multiple-environment trials. ANOVA can be used to estimate the size of genotype and G×E variance components. When running cultivar trials, genotypes are often considered to be fixed effects and environments random. However, for the purpose of estimating breeding values using best linear unbiased prediction (BLUP), often genotypes are considered to be random and environments fixed. Some may consider genotypes a random effect, provided that the objective is to select the best ones.^13^ If the G×E variance is significant, additional stability statistics can be calculated. However, ANOVA has limitations (i.e., the assumption of homogeneity of variance among environments) in its ability to explore the response of genotypes for G×E.^14^

A widely used approach for stability analysis is regression (*S*^*2*^_*d*_) of genotype performance relative to an environmental index derived from the average performance of all genotypes in each environment.^9,10,15,16^ Some researchers have reported problems with the regression method for evaluation of G×E patterns.^14,17–19^ The problems are of four types. First, the estimates of best fitted line have high error when only a few low- and high-yielding locations are included in the study.^20^ Second, the average of all genotypes evaluated in each environment (environmental index) is not independent of each genotype for that environment.^21^ Third, the errors associated with the slopes of genotypes are not statistically independent.^19^ Fourth, there is a required assumption of a linear relationship between interaction and environmental means when the actual responses of the genotypes to the environments are intrinsically multivariate.^20^ Shukla^22^ proposed an unbiased estimate of the variance (*σ*_*i*_^*2*^) of G×E plus an error term associated with genotype, in which a genotype with low *σ*_*i*_^*2*^ is regarded as stable. Kang's stability statistic (*YS*_*i*_) is nonparametric, using both trait mean (*M*) and *σ*_*i*_^*2*^, with equal weight on each. For Kang, genotypes with *YS*_*i*_ greater than the mean *YS*_*i*_ are stable.^23–25^

Multivariate analysis includes the genotype main effects plus genotypic x environment interaction effect (GGE) method and uses a graphical display for interpreting the results. These models are based on principal component (PC) analysis to help reveal structure in the data. The GGE biplot is constructed from the first two principal components (PC1 and PC2) that explain maximum variance, derived by singular value decomposition of a two-way (genotype-by-environment) data matrix.^26^

In this study, we were interested in components of watermelon fruit quality including lycopene content (mg kg^−1^), sugar content (%) and hollowheart resistance (rated 2–8, with 2=resistant, 8=susceptible). The objectives of this study were to (i) evaluate the G×E of watermelon genotypes, and (ii) identify watermelon genotypes with high stability for lycopene content, sugar content and hollowheart resistance.

## Materials and methods

### Germplasm and location

Forty genotypes of watermelon were evaluated for 3 years (2009, 2010 and 2011) and in eight locations across the southern United States. Locations were chosen to represent major watermelon production regions in the United States: North Carolina (Kinston, Clinton) and South Carolina (Charleston) in the east to Georgia (Cordele), Florida (Quincy), Oklahoma (Lane), and Texas (College Station) in the south to California (Woodland) in the west. Forty genotypes were chosen to represent new versus old releases, small versus large fruit size, round versus elongate fruit shape, striped versus solid rind pattern, anthracnose resistance versus susceptibility, eastern versus western adapted, and inbred versus hybrid type (Supplementary Table S1 and Supplementary Table S2).^27^ The 40 watermelon genotypes were categorized as inbred or hybrid based on information obtained from seed providers.^28^ Hybrids are identified with F_1_ after their name.

### Cultural practices

The experiment design was a randomized complete block with four replications, eight locations and three years. Seeds of each genotype were sown in 72-cell polyethylene flats in the greenhouses at North Carolina State University. The seedlings were transplanted by hand at the two-true-leaf stage. Missing or damaged transplants were replaced one week later.

Plots were planted on raised, shaped beds in rows on 3.1-m centers with plants 1.2 m apart (six plants/plot). The beds were irrigated with drip and covered with black polyethylene mulch. Production practices were according to the North Carolina Extension Service and Southeastern US 2009 Vegetable Crops handbook.^29,30^

### Data collection and traits

At each location and year, the 40 watermelon genotypes were evaluated for sugar content and hollowheart resistance. Ten genotypes were sampled at each location and year for lycopene content based on previous study^31^ and to represent a wide range of lycopene content and flesh color, including coral red, orange, and salmon yellow. These genotypes were 'Allsweet', 'Charleston Gray', 'Crimson Sweet', 'Hopi Red Flesh', 'Minilee', 'NC Giant', 'Sangria F1', 'Starbrite F1', 'Tendersweet Orange Flesh', and 'Yellow Crimson' (Supplementary Table S1). Genotypes 'Starbrite F1' and 'Sangria F1' are oval shape with coral red flesh; 'Allsweet' and 'Charleston Gray' are elongate and disease resistant; 'Crimson Sweet' has large round fruit and is widely grown around the world; 'Hopi Red Flesh' is low performing; 'NC Giant' has giant fruit, 'Minilee' has mini-sized fruit; 'Tendersweet Orange Flesh' has orange flesh and hollowheart susceptibility, and 'Yellow Crimson' has salmon yellow flesh color with low lycopene content. Data on the traits were not collected from Oklahoma in 2009, Georgia in 2010, and Florida in 2010 and 2011. Sugar content and hollowheart resistance data were not collected in South Carolina in 2009. Also, Florida and Texas did not record hollowheart resistance.

Three ripe fruit of each genotype per plot were individually sampled for lycopene content, sugar content and hollowheart resistance. Fruits were cut in half (stem to blossom), hollowheart defect was measured and a 100 g sample of watermelon flesh was taken from the center of the fruit (heart) for lycopene and sugar content. Width of the hollowheart defect (measure of hollowheart resistance) was recorded on the scale of 2 to 8 (2=0 mm or extreme resistance; 3=1–3 mm or high resistance; 4=3–5 mm or medium high resistance; 5=5–7 mm or medium resistance; 6=8–10 mm or medium less resistance; 7=10–12 mm or less resistance; and 8=>12 mm gap in flesh or no resistance to hollowheart defect).Sugar content, determined as total soluble solids, was measured as °Brix using a handheld digital refractometer (Atago 3810 PAL-1, Bellvue, WA, USA). At least 100 g of tissue from the heart (without seeds) placed into plastic bag and held on ice in an insulated cooler until return to laboratory.

Flesh samples were stored at −20 to −80 °C and samples were shipped on dry ice to the Plants for Human Health Institute, North Carolina Research Campus NCSU, Kannapolis, NC. Watermelon tissue was pureed using a homogenizer (Polytron pt 10/35, Kinematica, Bohemia, NY), then samples diluted with water (1:4 wt/vol) were transferred to a glass cuvette and absorbance measured using a Hunter Ultra Scan colorimeter (Hunter, NJ, USA). Lycopene was determined as mg kg^−1^ (p.p.m.) using the formula (absorbance_560nm_—absorbance _700nm)_×31.9, where 31.9 represents the slope derived from plotting sample colorimeter values against the same samples extracted with hexane and run on spectrophotometer, with an *R*^2^ of 0.93 (Shimazdu UV-160), following the method of Davis *et al*.^32^

### Data analysis

Data were analyzed for genotype, environment and G×E interactions using the SASGxE^27,33,34^ and RGxE^34,35^ programs. SASGxE and RGxE programs compute genotype stability statistics (univariate and multivariate), descriptive statistics, variance analysis and location value statistics using SAS and R programming languages, respectively. SASGxE and RGxE programs are freely available at http://cuke.hort.ncsu.edu/cucurbit/wehner/software.html.

Years, locations, replications, and genotypes were analyzed as random effects. Estimates and significance of random effects were computed using RGxE. Random effect model was fit using lmer() function of lme4 (linear mixed effects models) package,^36^ which is able to deal with unbalanced data. The significance of random effects was computed using likelihood ratio test to attain *P*-values. Likelihood is the probability of the data given a model. The logic of the likelihood ratio test is to compare the likelihood of two models with each other using restricted maximum likelihood (REML) methodology. The model without the factor that you are interested in (null model) is compared with model with the factor that you are interested in (full model) using anova() function. It gives a Chi-Square value, the associated degrees of freedom and *P*-value. According to Wilk’s theorem, the negative two times the log likelihood ratio of two models approaches a *χ*^2^ distribution with *k* degrees of freedom, where *k* is number of random effects tested.^37^ If G×E interactions were significant, additional statistics were calculated to determine the stability of each genotype over the 21 environments (location x year combinations).

RGxE was used to compute univariate stability statistics (regression slope (*b*_*i*_), deviation from regression (*S*^2^_*d*_), Shukla’s stability variance (*σ*_*i*_^2^), and Kang’s yield-stability statistics (*YS*_*i*_)), and BLUP for genotypes. Regression slope (*b*_*i*_) and deviation from regression (*S*^2^_*d*_); Shukla’s stability variance (*σ*_*i*_^2^) and Kang’s yield-stability statistics (*YS*_*i*_); and best linear unbiased predictor (BLUP) for genotypes were computed using lm() function of R;^38^ stability.par() function of the agricolae package;^39^ and ranef() function of lme4 package,^36^ respectively. Tests for significance were derived using a *t*-test for each *b*_*i*_ and an *F* test for each *S*^2^_*d*_ for statistical differences from one and zero, respectively, at 0.05, 0.01 and 0.001 levels of probability.

SASGxE provided R code that is ready to use in R statistical software^38^ for the analysis of multivariate stability statistics (GGE biplot). GGE biplot analysis was computed using the 'GGEBiplotGUI' package,^40^ with the support in the helper application ‘RStudio’^41^ in R statistical software. GGE biplot analysis was used to visually assess the presence of G×E interaction and rank genotype based on stability and mean.^12,26^ Input data of GGE biplot analysis consisted of G×E matrix (2×2) of BLUP values. BLUPs are estimates of random effects and account for missing data. Hereafter, the BLUP-GGE is used to represent the term GGE.

## Results

The analysis of variance identified significant environment (E), genotype (G), and G×E effects for all the evaluated traits (Table 1).

### Polygon view of BLUP-GGE biplot

**Figure 2:**
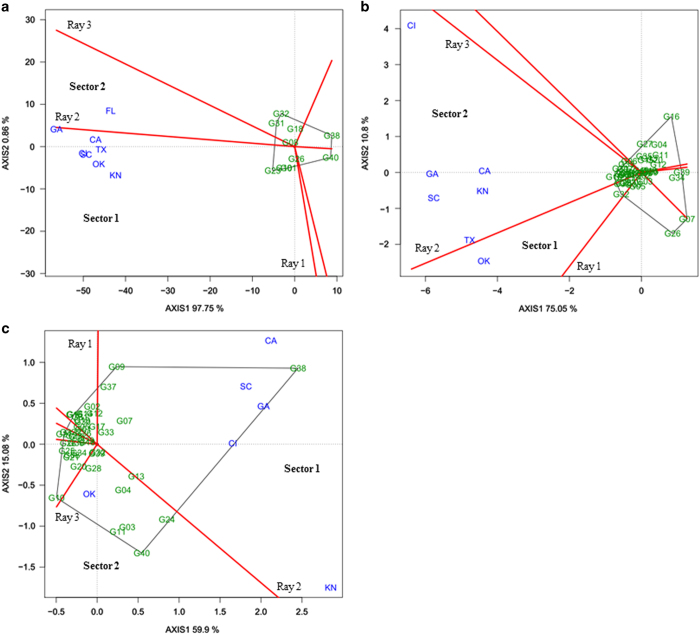
The polygon (which-won-where) view of BLUP-genotype main effects plus genotypic×environment interaction effect (BLUP-GGE) biplot for lycopene (mg kg^−1^) (**a**) of 10 watermelon genotypes, and sugar (°Brix) (**b**) and hollowheart resistance (**c**) of 40 watermelon genotypes tested in 3 years and 8 locations. The biplots were based on Scaling=0, Centering=0, and SVP=2. Key to the labels of genotype is presented in Supplementary Table S1 and location is CA, Woodland, CA; CL, Clinton, NC; FL, Quincy, FL; GA, Cordele, GA; KN, Kinston, NC; OK, Lane, OK; SC, Charleston, SC; TX, College Station, TX.

The polygon (which-won-where) view of the BLUP-GGE biplot divides the biplot into sectors using perpendicular lines (rays) that pass from the polygon sides (Figure 2). The polygon is drawn by joining the most extreme genotypes in the biplot. If environments fall into different sectors, then different genotypes won in different sectors, and a crossover G×E pattern exists. The winning genotype for an environment or set of environments in a sector is the vertex genotype. Conversely, if all environments fall into a single sector, a single genotype had the highest yield in all environments. The vertex genotype in a sector where no environment is present is considered to be a poor performer in all test environments. Genotypes within the polygon were less responsive to location than the vertex genotypes. A polygon view of the GGE biplot explained 99, 86 and 75% of the genotype and G×E variation for the lycopene content, sugar content and hollowheart resistance, respectively (Figures 2a–c). All three quality traits had environments grouped in two sectors with different winning genotypes (vertex genotype) in each (Figures 2a–c). This confirms the existence of G×E for lycopene content, sugar content and hollowheart resistance. (Figures 2a–c). Genotype main effects plus genotype x location interaction effect (GGL) biplots for individual year were constructed and showed that location grouping varied across years. Results of GGL biplots are presented in Table 2 and Supplementary Material (Supplementary Figure 1, Supplementary Figure 2 and Supplementary Figure 3).

### Genotype BLUPs

The significant G×E shows the need for evaluation of watermelon genotypes for stability for lycopene content, sugar content and hollowheart resistance. Estimates of genotype (random effect) performance for lycopene content ranged from 8.76 to 52.15 mg kg^−1^ (Table 3). Among red fleshed watermelons, highest lycopene content was in 'Minilee' and lowest was in 'Hopi Red Flesh'. For the non-red fleshed watermelons, 'Tendersweet Orange Flesh' and 'Yellow Crimson' had low lycopene content (Table 3). Similarly, sugar content ranged from 8.47 to 12.02 °Brix. Highest sugar content was measured in 'Crimson Sweet', 'Legacy', 'Regency F1', 'Royal Flush F1', 'Allsweet', 'Graybelle' and 'Sangria F1'. Lowest sugar content was for 'Carolina Cross #183', 'Navajo Sweet', 'Stone Mountain', 'Tom Watson', and 'Golden Midget' (Table 3). Hollowheart susceptibility was found in 'Tendersweet Orange Flesh', 'Mountain Hoosier', 'Yellow Crimson', 'Big Crimson' and 'Early Canada' with rating of 4 (3–5 mm wide gap in flesh). The remaining genotypes were resistant (rating of 3), with 'Minilee' the most resistant.

### Regression slope

According to Eberhart and Russell,^10^ a *b*_*i*_ approximating unity along with *S*^2^_*d*_ near zero indicate stability. The *b*_*i*_ value for lycopene content, sugar content and hollowheart resistance ranged from 0.144 to 2.132, −0.07 to 2.67 and −0.51 to 5.14, respectively (Supplementary Tables S3). For lycopene content, the *b*_*i*_ value for many genotypes was close (*P*>0.01) to unity, except for 'Allsweet', 'Tendersweet Orange Flesh' and 'Yellow Crimson' (Table 3 and Supplementary Table S3). Similarly, *b*_*i*_ for sugar content was significantly different from unity for 'Black Diamond', 'Carolina Cross#183', 'Charleston Gray', 'Congo', 'Desert King', 'Early Arizona', 'Georgia Rattlesnake', 'Legacy', 'Royal Flush F1', 'Sangria F1', 'Starbrite F1', and 'Stone Mountain' (Table 3 and Supplementary Table S4). 'Stone Mountain' had a negative *b*_*i*_ value. For hollowheart resistance, 'Calsweet', 'Georgia Rattlesnake', 'Mickylee', 'Minilee', 'Peacock WR-60' and 'Sangria F1' had negative *b*_*i*_ value and were significantly different from unity (Table 3 and Supplementary Table S5). Conversely, 'Early Arizona', 'Early Canada', 'Golden Midget' and 'Tendersweet Orange Flesh' had positive slope and were significantly different from unity for hollowheart resistance.

### Deviation from regression and Shukla’s stability variance

Except for 'Crimson Sweet' and 'Tendersweet Orange Flesh', all genotypes evaluated for lycopene content had significant *S*^2^_*d*_ and non-significant *σ*_*i*_^2^ (Table 3 and Supplementary Table S3). Genotypes 'Crimson Sweet' and 'Tendersweet Orange Flesh' had non-significant *S*^2^_*d*_ and lowest *σ*_*i*_^2^ for lycopene content. The top 12 genotypes with high sugar content (>11.0 °Brix) were 'Crimson Sweet', 'Legacy', 'Regency F1', 'Royal Flush F1', 'Allsweet', 'Graybelle', 'Sangria F1', 'Calsweet', 'Starbrite F1', 'Fiesta F1', 'Sugarlee', and 'Quetzali' All had non-significant *σ*_*i*_^*2*^ (Table 3 and Supplementary Table S4). Among these estimated high sugar content genotypes, three hybrids ('Royal Flush F1', 'Sangria F1' and 'Starbrite F1') and three inbreds ('Legacy', 'Allsweet' and 'Minilee') had significant *S*^*2*^_*d*_. Similarly, the top 12 genotypes with high hollowheart resistance (rating<3.20) were all inbreds, including 'NC Giant', 'Mickylee', 'Peacock WR-60', 'Crimson Sweet', 'Minilee', 'Georgia Rattlesnake', 'Legacy', 'Golden Midget', 'Navajo Sweet', 'King & Queen', 'Stone Mountain' and 'Sugar Baby'. Among these inbreds, all had non-significant *σ*_*i*_^2^, and genotypes 'NC Giant', 'Georgia Rattlesnake', 'Golden Midget' and 'King & Queen' had significant *S*^2^_*d*_ (Table 3 and Supplementary Table S5).

### Kang’s stability statistics

According to *YS*_*i*_, genotypes with *YS*_*i*_ higher than the mean *YS*_*i*_ are stable. For hollowheart resistance lower value is desired, *YS*_*i*_ lower than the mean *YS*_*i*_ are stable. The mean *YS*_*i*_ for lycopene content, sugar content and hollowheart resistance was 6.4, 20 and 19, respectively. According to *YS*_*i*_, the genotypes that were stable for all three quality traits (lycopene content, sugar content and hollowheart resistance) were 'Charleston Gray', 'Crimson Sweet', and 'Starbrite F1' (Table 3).

### Mean versus stability and genotype comparison with ideal genotype views of BLUP-GGE biplot

**Figure 3:**
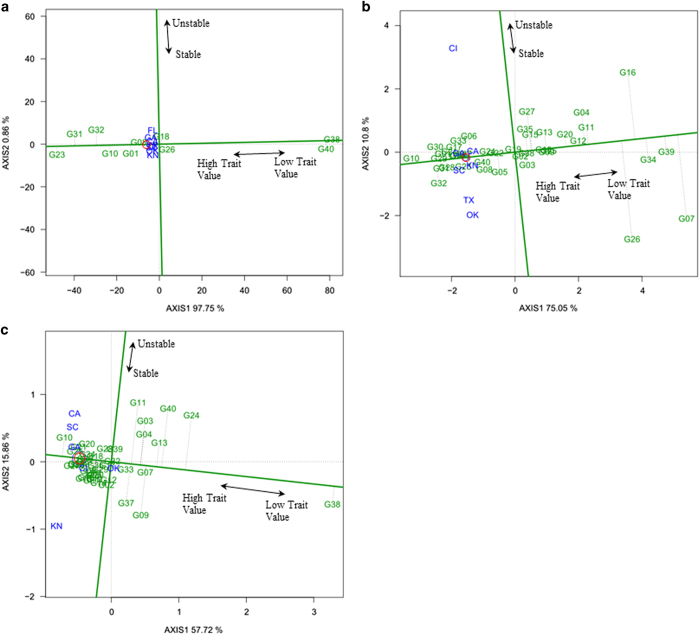
The mean versus stability view of BLUP-genotype main effects plus genotypic×environment interaction effect (BLUP-GGE) biplot for lycopene (mg kg^−1^) (**a**) of 10 watermelon genotypes, and sugar (°Brix) (**b**) and hollowheart resistance (**c**) of 40 watermelon genotypes tested in 3 years and 8 locations. The biplots were based on Scaling=0, Centering=2, and SVP=1. The ideal genotype is represented by a circle on average environment coordinate (AEC)-abscissa which passed through biplot origin. For hollowheart defect, high trait value represent no or minimum hollowheart defect present (**c**). Key to the labels of genotype is presented in Supplementary Table S1 and location is CA, Woodland, CA; CL, Clinton, NC; FL, Quincy, FL; GA, Cordele, GA; KN, Kinston, NC; OK, Lane, OK; SC, Charleston, SC; TX, College Station, TX.

The average environment coordinate (AEC) view based on genotype-focused singular value partitioning (SVP=1) and BLUP values can be referred to as the 'BLUP versus stability' view (equivalent of 'mean versus stability' view proposed by Yan *et al.*^42^) of BLUP-GGE biplot. This view permits genotype comparisons based on estimates of genotype performance and stability across environments within a mega-environment. The ‘mean versus stability’ view of BLUP-GGE biplot explained 99%, 85 and 74% of genotypic and G×E variation for the lycopene content, sugar content and hollowheart resistance, respectively (Figures 3a–c). The arrow shown (red circle is marked on the head of arrow) on the AEC abscissa points in the direction of higher trait performance of genotypes, and ranks the genotypes with respect to trait performance. Thus, 'Minilee' (G23) had the highest estimate of lycopene content, and 'Tendersweet Orange Flesh' (G38) and 'Yellow Crimson' (G40) had the lowest (Figure 3a). Similarly, 'Crimson Sweet' (G10) had the highest estimate of sugar content and hollowheart resistance (Figures 3b and c). Genotypes 'Tendersweet Orange Flesh' (G38) and 'Yellow Crimson' (G07) had the lowest estimate of estimate of sugar content and hollowheart resistance (Figured 3b and c).

The stability of each genotype was explored by its projection onto the AEC vertical axis. The most stable genotype was located almost on the AEC horizontal axis and had a near-zero projection onto the AEC vertical axis. All 10 genotypes evaluated in the study for lycopene content were close to the AEC horizontal axis and had minimum or no vertical projection, indicating stability (Figure 3a). Genotypes with low sugar content and high hollowheart resistance had a high vertical projection on the AEC horizontal axis, and genotypes situated left of biplot origin were less stable (Figures 3b and c).

The ideal genotype has both high trait mean and stable performance. An ideal genotype is represented by a circle on the head of the arrow on the AEC abscissa (horizontal axis) (Figures 3a–c). For lycopene content, 'Minilee' (G23), 'Sangria F1' (G31), 'Starbrite F1' (G32), 'Crimson Sweet' (G10) and 'Allsweet' (G01) were best (Figure 3a). Similarly, for sugar content 'Crimson Sweet' (G10), 'Regency F1' (G29), 'Royal Flush F1' (G30), 'Starbrite F1' (G32), 'Sangria F1' (G31), and 'Quetzali' (G28) were best (Figure 3b). For hollowheart resistance 'Crimson Sweet' (G10), 'NC Giant' (G25), 'Legacy' (G21), 'Peacock WR-60' (G27), 'King & Queen' (G20), 'Sugar Baby' (G35), 'Stone Mountain' (G34), and 'Minilee' (G23) were best (Figure 3c).

**Figure 4:**
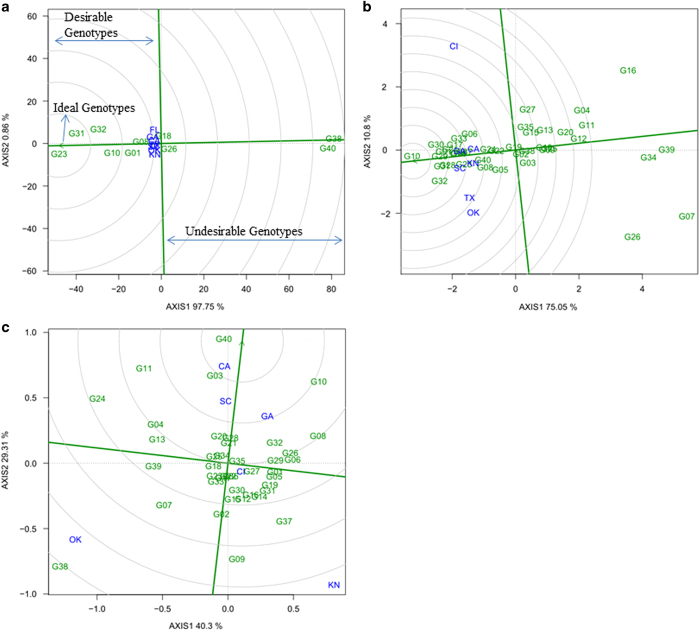
The genotypes comparison with ideal genotype view of BLUP-genotype main effects plus genotypic×environment interaction effect (BLUP-GGE) biplot for lycopene (mg kg^−1^) (**a**) of 10 watermelon genotypes, and sugar (°Brix) (**b**) and hollowheart resistance (**c**) of 40 watermelon genotypes tested in 3 years and 8 locations. The biplots were based on Scaling=0, Centering=2, and SVP=1. An ideal genotype is represented by circle within innermost concentric circles on average environment coordinate (AEC)-abscissa which passed through biplot origin. Key to the labels of genotype is presented in Supplementary Table S1 and location is CA, Woodland, CA; CL, Clinton, NC; FL, Quincy, FL; GA, Cordele, GA; KN, Kinston, NC; OK, Lane, OK; SC, Charleston, SC; TX, College Station, TX.

The 'comparison with ideal genotype' view of BLUP-GGE biplot has concentric circles with the ideal genotype in the inner circle and the head of the arrow is the center of the circle (the arrow is highlighted) (Figures 4a–c). The genotypes grouped in the inner circle (ideal genotypes) are more desirable than the others. Thus, 'Minilee' (G23) and 'Sangria F1' (G31) were the most desirable genotypes for lycopene content (Figure 4a). For sugar content and hollowheart resistance, 'Crimson Sweet' (G10) and 'Yellow Crimson' (G40), respectively were desirable (Figures 4b and c). The genotypes in the second inner circle are next most desirable. The results of 'comparison with ideal genotype' view of BLUP-GGL biplots are presented in Table 2 and Supplementary Material (Supplementary Figure 7, Supplementary Figure 8 and Supplementary Figure 9).

## Discussion

Previous studies of the stability of yield and yield components in watermelon identified four genotype stability-performance combinations: high stability-high yield, high stability-low yield, low stability-high yield, and low stability-low yield.^27,43,44^ Based on marketable yield, Dia et al.^27^ grouped 40 watermelon genotypes evaluated in this study into high (top 10), mid-high (11–20), mid-low (21–30) and low (bottom 10) for yield. The high-yielding hybrids 'Starbrite F1', 'Stars-N-Stripes F1', 'Fiesta F1', and 'Regency F1' also had high sugar content and hollowheart resistance (Table 3). The high-yielding inbreds 'Big Crimson', 'Stone Mountain', 'Calhoun Gray', and 'Legacy' had mid-high to high sugar content and moderate to high hollowheart resistance (Table 3). Conversely, mid-high to mid-low yielding hybrids 'Sangria F1' and 'Royal Flush F1' and inbreds 'Calsweet' and 'Sugarlee' had high sugar content and hollowheart resistance. The findings suggest that yield was not correlated with fruit quality. Plant breeders may want to use these moderate yield inbreds to develop new cultivars with high sugar content and hollowheart resistance.

Of the 10 genotypes evaluated for lycopene content, those with high yield were 'Starbrite F1' and 'Yellow Crimson', followed by 'Sangria F1', 'Tendersweet Orange Flesh' and 'NC Giant', followed by 'Charleston Gray' and 'Allsweet', followed by 'Hopi Red Flesh', 'Crimson Sweet' and 'Minilee'.^27^ Genotypes with high marketable yield ('Starbrite F1' and 'Yellow Crimson') ranged from high to low for lycopene content, indicating that progress can be made in combing quality with yield (Table 3). That pattern was consistent for sugar content and hollowheart resistance. Estimates of genotype (random effect) performance for lycopene content, sugar content and hollowheart resistance used the restricted maximum likelihood/BLUP (based on mixed models) methodology. BLUPs tend to be ‘shrunk’ towards the population mean relative to their fixed effects, and predict genotype performance more accurately than mean or LS mean alone. Therefore, plant breeders can use BLUPs as selection criteria to predict genotype performance.

The visualization of 'which won where' pattern of BLUP-GGE biplot reveals the existence of a crossover pattern of G×E and, thus, different mega-environments among the watermelon growing regions. Each mega-environment is a group of growing areas that are similar in terms of genotype response, and that show a repeatable relative performance of crop genotypes across years.^45,46^ However, a definitive conclusion on existence of mega-environment should be based on repeatable crossover patterns. Yearly BLUP-GGL biplot for lycopene content, sugar content and hollowheart resistance suggested that test locations had different winning genotypes that were not repeatable across years (Table 2, Supplementary Figure 1, Supplementary Figure 2 and Supplementary Figure 3). It appears that the G×E that causes the crossovers among winning genotypes cannot be exploited or converted into G.^42^ Therefore, for lycopene content, sugar content and hollowheart resistance the target environment is essentially a single but complex mega-environment. Watermelon breeders should select widely adapted genotypes for the whole region based on both mean performance and stability analysis using multi-environment trial data.

Based on average ranking generated from multiple stability measures (BLUP, *b*_*i*_, *S*^2^_*d*_, *σ*_*i*_^2^, *YS*_*i*_ and 'mean versus stability' view of BLUP-GGE biplot), watermelon genotypes could be classified into three categories (Table 4). Category 1 was genotypes having high trait performance and high stability. These genotypes are widely adapted across diverse environments. Among all evaluated genotypes, 'Crimson Sweet' was the most desirable since it had high lycopene content, sugar content and hollowheart resistance, and high stability. For sugar content, inbreds 'Crimson Sweet', 'Legacy', 'Allsweet', 'Graybelle', and 'Quetzali' and hybrids 'Regency F1' and 'Fiesta F1' had high trait performance and stability. The top six genotypes for hollowheart resistance were inbreds and all had high stability. Those were 'NC Giant', 'Peacock WR-60', 'Mickylee', 'Crimson Sweet', 'Minilee' and 'Legacy'.

Category 2 genotypes exhibited high trait performance but low stability, so these genotypes are suited for specific environments. This category includes genotypes 'Sangria F1', 'Calsweet', 'Starbrite F1'¸ 'Sugarlee', and 'Stars-N-Stripes F1' for sugar content; 'King & Queen', 'Sugarlee' and 'Charleston Gray' for hollowheart resistance; and 'Minilee', 'Starbrite F1' and 'Allsweet' for lycopene content. Category 3 genotypes had low trait performance and high stability. These genotypes are suitable for breeding 'health smart' varieties with low sugar content and high lycopene content. Inbreds 'Stone Mountain', 'Tom Watson' and 'Navajo Sweet' had low sugar, low hollowheart defect and high stability. For high lycopene and stability, inbreds 'Crimson Sweet' and 'Minilee' were best. ‘Minilee’ had high lycopene content even though it does not have the gene *Y*^*Scr*^ (scarlet red flesh), so should be investigated as an independent source of high lycopene. Category 1, 2 and 3 genotypes were spread over the range for marketable yield, fruit count, percentage cull fruit, percentage early fruit and fruit size.^27^

The 'mean versus stability' view of BLUP-GGL biplot revealed a unique pattern of stability across years based on projection onto the AEC vertical axis (Table 2, Supplementary Figure 4, Supplementary Figure 5 and Supplementary Figure 6). For lycopene content, almost all genotypes had minimum projection on the AEC abscissa. Conversely, for sugar content and hollowheart resistance, genotypes that were estimated for low trait performance (right of biplot origin) had high projection. Therefore, they were relatively less stable. The estimate of trait performance varied but stability pattern was fairly consistent across years. In addition to three quality traits evaluated in this study, a wide range of phenotypic expression existed among Category 1, 2 and 3 watermelon genotypes (Supplementary Table 2). Therefore, researchers can introgress genes into elite inbreds to make better hybrids with high performance and high stability. Inbreds 'Crimson Sweet' and 'Legacy' would be good parents in breeding since they had high lycopene content and sugar content, along with hollowheart resistance, and high stability (Note: 'Legacy' was not evaluated for lycopene). However, inbred 'Sugarlee' and hybrid 'Stars-N-Stripes F1' had high trait mean but low stability. Stability is meaningful when it is associated with high yield. Therefore, we have developed a stability chart based on marketable yield^27^ and average ranking generated from different stability measures for yield attributes^27^ and quality traits of 40 watermelon genotypes tested for 3 years and 8 locations (Table 4). See http://cuke.hort.ncsu.edu/cucurbit/wmelon/wmelonmain.html.

For the development of stable genotypes for lycopene content, sugar content and hollowheart resistance, the pattern of G×E interaction varied among univariate and multivariate stability statistics. Based on the results, it appears possible to breed stable genotypes with either high or low quality traits. The genotype with highest performance and greatest stability for fruit quality was the inbred line, 'Crimson Sweet'.

## Figures and Tables

**Table 1:** Variance analysis for lycopene content (mg kg^−1^) of 10 watermelon genotypes, and sugar content (°Brix) and hollowheart resistance of 40 watermelon genotypes tested in 3 years and 8 locations

*Source*	*Estimate*	*s.d.*	χ*^2^ probability*
	Lycopene		
Location (L)	0.48	0.69	NS
Year (Y)	0.004	0.002	NS
Environment (L×Y)	11.47	3.38	***
Replication within E	4.05	2.01	***
Genotype (G)	233.55	15.2	***
G×L	0.001	0.001	NS
G×Y	3.62	1.90	**
G×E	10.64	3.26	***
Pooled error	30.37	5.51	
	
	Sugar		
Location (L)	0.19	0.44	NS
Year (Y)	0.005	0.0008	NS
Environment (L×Y)	0.18	0.43	***
Replication within E	0.13	0.37	***
Genotype (G)	0.61	0.78	***
G×L	0.04	0.19	NS
G×Y	0.06	0.26	*
G×E	0.32	0.56	***
Pooled error	1.01	1.00	
	
	Hollowheart		
Location (L)	0.001	0.0004	NS
Year (Y)	0.002	0.004	NS
Environment (L×Y)	0.02	0.13	**
Replication within E	0.005	0.07	*
Genotype (G)	0.07	0.25	***
G×L	0.02	0.13	NS
G×Y	0.003	0.02	NS
G×E	0.12	0.33	***
Pooled error	0.35	0.59	

Abbreviation: NS, non-significant.

*, ** and *** significant at 0.05, 0.01 and 0.001 levels of probability, respectively.

**Table 2:** Summary of yearly performance of 10 watermelon genotypes for lycopene content and 40 watermelon genotypes for sugar content (°Brix) and hollowheart resistance tested in 3 years and 8 locations based on polygon (which-won-where), mean versus stability and genotypes comparison with ideal genotype view of BLUP-genotype main effects plus genotypic×location interaction effect (BLUP-GGL) biplot

*Trait*	*Parameter*	*Which-won-where view*		
		*Year 2009*	*Year 2010*	*Year 2011*
	Sector1 winning genotype	G23 (CA, CI, GA, FL)^a^	G31 (CA, CI, KN, OK, SC, TX)	G23 (CA, CI, KN, OK, SC, TX)
Lycopene	Sector2 winning genotype	G31 (KN, SC, TX)		G31 (GA)
Sugar	Sector1 winning genotype	G30 (CI, KN)	G30 (CA, KN, OK, SC, TX)	G10 (CA, CI, GA, KN, TX)
	Sector2 winning genotype	G29 (CA)	G36 (CI)	
	Sector3 winning genotype	G31 (GA)		
Hollowheart resistance	Sector1 winning genotype	G40 (CI, GA, KN)	G08 (OK)	G03 (KN)
	Sector2 winning genotype	G16 (CA)	G38 (CA, CI, KN, SC)	G38 (CA, CI, GA, SC)
				
		*Mean versus stability view*		
				
		*Year 2009*	*Year 2010*	*Year 2011*
				
Lycopene	Stable and high performance	G31>G32>G10=G23>G01	G31>G10>G23>G32=G08	G23>G18=G10>G31=G32
Sugar	Stable and high performance	G33>G24=G10=G29>G06	G30>G28=G32>G29=G14=G31	G10>G01>G36>G18=G31=GG29
Hollowheart resistance	Stable and high performance	G27>G17>G20>G18>G25=G22	G18>G15>G22>G23	G16=G01>G10
				
		*Genotypes comparison with ideal genotype view*		
				
		*Year 2009*	*Year 2010*	*Year 2011*
				
Lycopene	Ideal genotypes	G31, G23, G32	G23, G31	G23, G31, G32
Sugar	Ideal genotypes	G33	G32, G28, G30	G10, G01
Hollowheart resistance	Ideal genotypes	G01, G28	G11, G28, G03	G24, G03, G04, G18

a Winning genotype within group or single environment.

**Table 3:** Mean (BLUP) and significance value of regression coefficient (*b*_*i*_), deviation from regression (*S*^2^_*d*_), Shukla’s stability variance (*σ*_*i*_^2^), and Kang’s stability statistics (*YS*_*i*_) for lycopene content (mg kg^−1^) of 10 watermelon genotypes, and sugar content (°Brix) and hollowheart resistance of 40 watermelon genotypes tested in 3 years and 8 locations

*Genotype*	*Lycopene*					*Sugar*					*Hollowheart*				
	*BLUP (mg kg*^*−1*^)	*b*_*i*_,	*S*^*2*^_*d*_	*σ*_*i*_^*2*^	*YS*_*i*_	*BLUP (*°*Brix)*	*b*_*i*_,	*S*^*2*^_*d*_	*σ*_*i*_^*2*^	*YS*_*i*_	*BLUP (2–8 scale)*	*b*_*i*_,	*S*^*2*^_*d*_	*σ*_*i*_^*2*^	*YS*_*i*_
AU-Jubilant						10.53	.	.	.	√	3.21	.	*	.	√
Allsweet^a^	41.55	***	***	.	√^b^	11.69	.	*	.	.	3.24	.	*	.	√
Big Crimson						10.59	.	.	.	.	3.56	.	*	.	√
Black Diamond						10.16	*	.	.	.	3.42	.	***	.	√
Calhoun Gray						10.85	.	.	.	√	3.20	.	.	.	.
Calsweet						11.61	.	.	.	√	3.18	*^c^	.	.	.
Carolina Cross#183						8.47	*	***	**	.	3.39	.	.	.	√
Charleston Gray^a^	38.60	.	*	.	√	10.82	*	*	.	√	3.17	.	***	.	√
Congo						10.45	**	.	.	.	3.41	.	**	**	√
Crimson Sweet^a^	43.83	.	.	.	√	12.02	.	.	.	√	3.05	.	.	.	√
Desert King						10.15	***	**	.	.	3.41	.	**	**	√
Early Arizona						10.05	*	**	.	.	3.28	*	.	.	√
Early Canada						10.51	.	*	.	.	3.53	*	.	.	√
Fiesta F1						11.57	.	.	.	√	3.19	.	*	.	.
Georgia Rattlesnake						10.59	***	**	.	.	3.09	**^c^	*	.	.
Golden Midget						9.64	.	***	**	.	3.11	***	***	.	.
Graybelle						11.65	.	.	.	√	3.31	.	.	.	√
Hopi Red Flesh^a^	34.31	.	***	.	.	10.51	.	***	.	.	3.20	.	***	.	.
Jubilee						10.83	.	.	.	√	3.20	.		.	√
King & Queen						10.13	.	.	.	.	3.12	.	***	.	.
Legacy						11.77	*	*	.	√	3.09	.	.	.	.
Mickylee						10.89	.	.	.	√	3.03	**^c^	.	.	.
Minilee^a^	52.15	.	**	.	√	11.47	.	.	.	√	3.06	***^c^	.	. c	.
Mountain Hoosier						11.29	.	.	.	√	3.72	.	.	.	√
NC Giant^a^	33.58	.	***	.	.	10.42	.	.	.	.	3.01	.	***	.	.
Navajo Sweet						9.10	.	.	**	.	3.11	.	.	.	.
Peacock WR-60						10.9	.	.	**	.	3.03	***^c^	.	.	.
Quetzali						11.53	.	.	.	√	3.27	.	.	.	√
Regency F1						11.71	.	.	.	√	3.25	.	.	.	√
Royal Flush F1						11.70	***	**	.	√	3.16	.	.	.	.
Sangria F1^a^	48.62	.	*	.	√	11.65	*	**	.	√	3.18	*^c^	*	.	.
Starbrite F1^a^	44.76	.	***	.	√	11.58	*	**	.	√	3.37	.	.	.	√
Stars-N-Stripes F1						11.36	.	.	.	√	3.32	.	.	.	√
Stone Mountain						9.14	***^c^	***	.	.	3.12	.	.	.	.
Sugar Baby						10.60	.	.	.	.	3.13	.	.	.	.
Sugarlee						11.57	.	.	.	√	3.16	.	.	.	.
Sweet Princess						11.38	.	.	.	√	3.42	.	.	.	√
Tendersweet OF^a^	8.76	*	.	.	.	10.79	.	.	.	.	4.77	**	***	.	√
Tom Watson						9.17	.	.	.	.	3.17	.	.	.	.
Yellow Crimson^a^	8.98	**	**	.	.	11.19	.	.	.	√	3.70	.	**	.	√

*, ** and *** significantly different from unity for the regression coefficients or slope (*b*_*i*_) and from zero for the deviation from regression (*S*^2^_*d*_) and Shukla’s stability variance (*σ*_*i*_^2^) at 0.05, 0.01 and 0.001 levels of probability, respectively.

a Indicate genotypes sampled for lycopene analysis.

b √ indicates stable according to Kang stability statistics (*YS*_*i*_).

c Indicates negative slope.

**Table 4:**
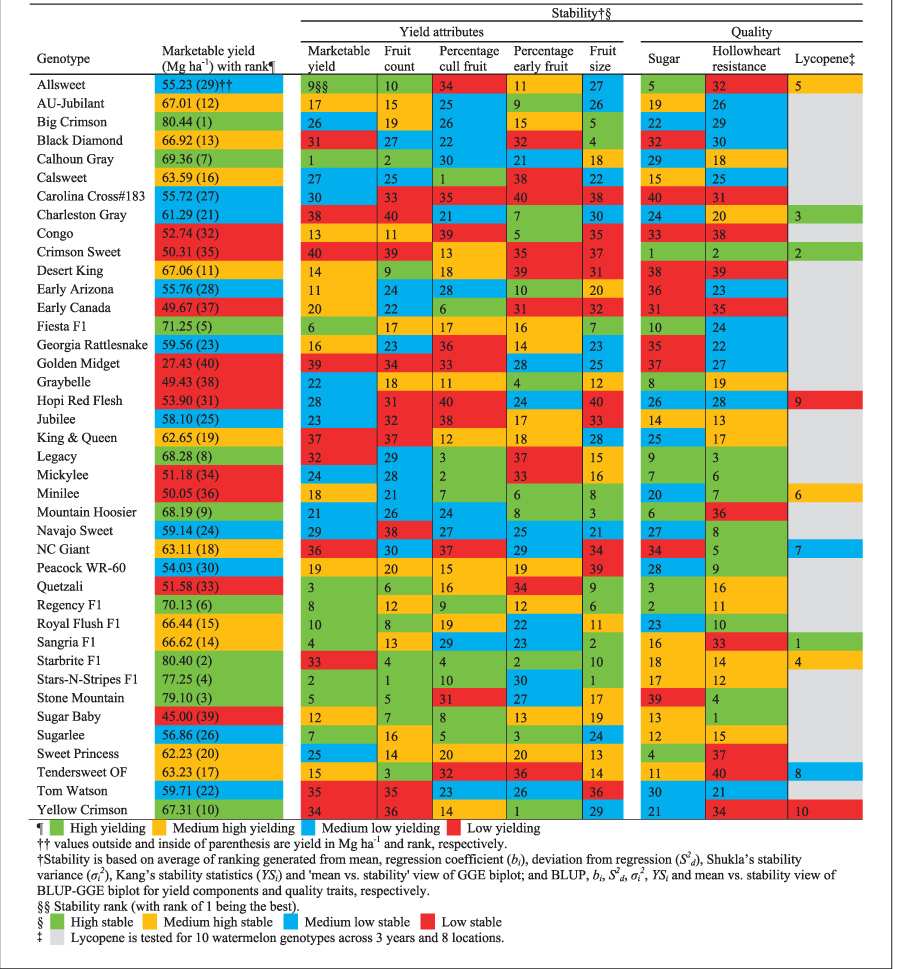
Stability chart to identify high yielding and stable watermelon genotype for yield components (marketable yield, fruit count, percentage cull fruit, percentage early fruit and fruit size^27^ and quality traits (lycopene, sugar and hollowheart resistance) based on 40 watermelon genotypes tested in 3 years and 8 locations
