# Supplementary material for: Stability of fruit quality traits in diverse watermelon cultivars tested in multiple environments
Source: Hortic Res. 2016 Dec 21;3:16066–. doi: 10.1038/hortres.2016.66 (PMC5174159; doi:10.1038/hortres.2016.66)
Supplement: Supplementary Information [file hortres201666-s1.doc]

**Supplement Material Description**

The supplemental tables provide a listing of pedigree, phenotypes, and stability parameters (*b_i_* , *S^2^_d_*, *σ_i_^2^*, and *YS_i_*) for lycopene, and sugar and hollowheart defect of 10 and 40 watermelon genotypes, respectively, evaluated in the study. Similarly, supplemental figures provide yearly BLUP-GGE biplots (which-won-where or polygon view, mean vs. stability view, and genotype comparison with ideal genotype view) for lycopene, and sugar and hollowheart resistance.

**Supplemental Table Captions:**

Supplemental Table 1. The 40 watermelon genotypes tested with pedigree information.

Supplemental Table 2. Fruit and seed traits for the 40 watermelon genotypes evaluated.

Supplemental Table 3. BLUP, regression coefficient (*b_i_*), deviation from regression (*S^2^_d_*), Shukla’s stability variance (*σ_i_^2^*), and Kang’s stability statistics (*YS_i_*) for lycopene of 10 watermelon genotypes tested in 3 year and 8 locations.

Supplemental Table 4. BLUP, regression coefficient (*b_i_*), deviation from regression (*S^2^_d_*), Shukla’s stability variance (*σ_i_^2^*), and Kang’s stability statistics (*YS_i_*) for sugar of 40 watermelon genotypes tested in 3 year and 8 locations.

Supplemental Table 5. BLUP, regression coefficient (*b_i_*), deviation from regression (*S^2^_d_*), Shukla’s stability variance (*σ_i_^2^*), and Kang’s stability statistics (*YS_i_*) for hollowheart resistance of watermelon genotypes tested in 3 year and 8 locations.

**Supplemental Figure Captions:**

Supplemental Figure 1. The polygon (which–won–where) view of BLUP-genotype main effects plus genotypic x location interaction effect (BLUP-GGL) biplot of 10 watermelon genotypes tested in 3 year (Panel A: 2009, Panel B: 2010, and Panel C: 2011) and 8 locations for lycopene. The biplots were based on Scaling = 0, Centering = 0, and SVP = 2.

Supplemental Figure 2. The polygon (which–won–where) view of BLUP-genotype main effects plus genotypic x location interaction effect (BLUP-GGL) biplot of 40 watermelon genotypes tested in 3 year (Panel A: 2009, Panel B: 2010, and Panel C: 2011) and 8 locations for sugar. The biplots were based on Scaling = 0, Centering = 0, and SVP = 2.

Supplemental Figure 3. The polygon (which–won–where) view of BLUP-genotype main effects plus genotypic x location interaction effect (BLUP-GGL) biplot of 40 watermelon genotypes tested in 3 year (Panel A: 2009, Panel B: 2010, and Panel C: 2011) and 8 locations for hollowheart resistance. The biplots were based on Scaling = 0, Centering = 0, and SVP = 2.

Supplemental Figure 4. The mean vs. stability view of BLUP-genotype main effects plus genotypic x location interaction effect (BLUP-GGL) biplot of 10 watermelon genotypes tested in 3 year (Panel A: 2009, Panel B: 2010, and Panel C: 2011) and 8 locations for lycopene. The biplots were based on Scaling = 0, Centering = 2, and SVP = 1. The ideal genotype is represented by a circle on average environment coordinate (AEC)-abscissa which passed through biplot origin.

Supplemental Figure 5. The mean vs. stability view of BLUP-genotype main effects plus genotypic x location interaction effect (BLUP-GGL) biplot of 40 watermelon genotypes tested in 3 year (Panel A: 2009, Panel B: 2010, and Panel C: 2011) and 8 locations for sugar. The biplots were based on Scaling = 0, Centering = 2, and SVP = 1. The ideal genotype is represented by a circle on average environment coordinate (AEC)-abscissa which passed through biplot origin.

Supplemental Figure 6. The mean vs. stability view of BLUP-genotype main effects plus genotypic x location interaction effect (BLUP-GGL) biplot of 40 watermelon genotypes tested in 3 year (Panel A: 2009, Panel B: 2010, and Panel C: 2011) and 8 locations for hollowheart resistance. The biplots were based on Scaling = 0, Centering = 2, and SVP = 1. The ideal genotype is represented by a circle on average environment coordinate (AEC)-abscissa which passed through biplot origin.

Supplemental Figure 7. The genotypes comparison with ideal genotype view of BLUP-genotype main effects plus genotypic x location interaction effect (BLUP-GGL) biplot of 10 watermelon genotypes tested in 3 year (Panel A: 2009, Panel B: 2010, and Panel C: 2011) and 8 locations for lycopene. The biplots were based on Scaling = 0, Centering = 2, and SVP = 1. An ideal genotype is represented by circle within innermost concentric circles on average environment coordinate (AEC)-abscissa which passed through biplot origin.

Supplemental Figure 8. The genotypes comparison with ideal genotype view of BLUP-genotype main effects plus genotypic x location interaction effect (BLUP-GGL) biplot of 40 watermelon genotypes tested in 3 year (Panel A: 2009, Panel B: 2010, and Panel C: 2011) and 8 locations for sugar. The biplots were based on Scaling = 0, Centering = 2, and SVP = 1. An ideal genotype is represented by circle within innermost concentric circles on average environment coordinate (AEC)-abscissa which passed through biplot origin.

Supplemental Figure 9. The genotypes comparison with ideal genotype view of BLUP-genotype main effects plus genotypic x location interaction effect (BLUP-GGL) biplot of 40 watermelon genotypes tested in 3 year (Panel A: 2009, Panel B: 2010, and Panel C: 2011) and 8 locations for hollowheart resistance. The biplots were based on Scaling = 0, Centering = 2, and SVP = 1. An ideal genotype is represented by circle within innermost concentric circles on average environment coordinate (AEC)-abscissa which passed through biplot origin.

Table S1. The 40 watermelon genotypes tested.

| ID | Genotype | Year of release | Pedigree |
| --- | --- | --- | --- |
| G1 | Allsweet^L^ | 1972 | [(Miles x Peacock) x Charleston Gray] |
| G2 | AU-Jubilant | 1985 | Jubilee x PI 271778 |
| G3 | Big Crimson | NA† | NA |
| G4 | Black Diamond | 1949 | Segregation within Cannonball or Black Diamond |
| G5 | Calhoun Gray^L^ | 1965 | Calhoun Sweet x Charleston Gray |
| G6 | Calsweet | NA | [(Miles x Peacock) x Charleston Gray] |
| G7 | Carolina Cross#183 | NA | NA |
| G8 | Charleston Gray | 1954 | [{(Africa 8 x Iowa Belle) x Garrison} x Garrison] x [(Hawkesbury x Leesburg) x Garrison] |
| G9 | Congo | 1949 | (African x Iowa Belle) x Garrison |
| G10 | Crimson Sweet^L^ | 1963 | (Miles x Peacock) x Charleston Gray |
| G11 | Desert King | NA | NA |
| G12 | Early Arizona | NA | NA |
| G13 | Early Canada | NA | NA |
| G14 | Fiesta F1 | 1991 | Unknown (Plant Variety Protection) |
| G15 | GeorgiaRattlesnake | 1870 |  |
| G16 | Golden Midget | 1959 | New Hampshire Midget x Pumpkin Rind |
| G17 | Graybelle | 1963 | Sugar Baby x Charleston Gray sister line |
| G18 | Hopi Red Flesh^L^ | NA | NA |
| G19 | Jubilee | 1963 | Africa 8, Iowa Belle, Garrison, Hawkesbury, and Leesburg |
| G20 | King & Queen | NA | NA |
| G21 | Legacy | 1997 | (Early Gray x Little Jubilee 4) x Verona |
| G22 | Mickylee | 1986 | Texas W5, Fairfax, Summit, and Graybelle |
| G23 | Minilee^L^ | 1986 | Texas W5, Fairfax, Summit, and Graybelle |
| G24 | Mountain Hoosier | NA | NA |
| G25 | Navajo Sweet | NA | NA |
| G26 | NC Giant^L^ | NA | NA |
| G27 | Peacock WR-60 | 1955 | Klondike R7 x Peacock |
| G28 | Quetzali | 1965 | NA |
| G29 | Regency F1 |  | Unknown (Plant Variety Protection) |
| G30 | Royal Flush F1 | 1995 | Unknown (Plant Variety Protection) |
| G31 | Sangria F1^L^ | NA | Unknown (Plant Variety Protection) |
| G32 | Starbrite F1^L^ | NA | Unknown (Plant Variety Protection) |
| G33 | Stars-N-Stripes F1 | NA | Unknown (Plant Variety Protection) |
| G34 | Stone Mountain | 1924 | NA |
| G35 | Sugar Baby | 1955 | Tough Sweets selection, inbred 13 years |
| G36 | Sugarlee | 1981 | Texas W5, Summit, Charleston Gray, Fairfax, Crimson Sweet, and Graybelle |
| G37 | Sweet Princess | 1967 | small-seeded Congo type x Charleston Gray |
| G38 | Tendersweet OF^L^ | NA | NA |
| G39 | Tom Watson | 1906 | NA |
| G40 | Yellow Crimson^L^ | NA | NA |

† Not available

^L^ Used for lycopene

Table S2. Traits and pedigrees for the 40 watermelon genotypes evaluated.

| Genotype |  | Fruit | |  | Rind | | |  | Seed | |  | Flesh color¶¶ |
| --- | --- | --- | --- | --- | --- | --- | --- | --- | --- | --- | --- | --- |
|  |  | Shape† | Size‡ |  | Color§ | Thickness¶ | Pattern# |  | Size†† | Color‡‡ |  |  |
| AU-Jubilant |  | L | M |  | LG | S | M |  | L | R |  | R |
| Allsweet^L^ |  | L | M |  | LG | N | N |  | S | R |  | R |
| Big Crimson |  | R | M |  | MG | M | N |  | M | R |  | R |
| Black Diamond |  | R | S |  | DB | M | S |  | L | R |  | R |
| Calhoun Gray |  | L | M |  | G | M | S |  | M | R |  | R |
| Calsweet |  | L | M |  | LG | T | N |  | S | B |  | S |
| Carolina Cross#183 |  | E | G |  | LG | T | W |  | L | W |  | R |
| Charleston Gray^L^ |  | L | L |  | G | T | R |  | M | R |  | R |
| Congo |  | L | M |  | DG | M | R |  | L | T |  | R |
| Crimson Sweet^L^ |  | R | M |  | LG | M | M |  | S | B |  | R |
| Desert King |  | O | S |  | SG | S | S |  | M | R |  | O |
| Early Arizona |  | O | S |  | SG | N | S |  | L | R |  | R |
| Early Canada |  | R | S |  | G | N | R |  | S | R |  | R |
| Fiesta F1 |  | L | M |  | LG | M | N |  | S | B |  | R |
| Georgia Rattlesnake |  | L | G |  | LG | M | W |  | L | R |  | R |
| Golden Midget |  | O | C |  | Y | N | S |  | L | R |  | R |
| Graybelle |  | G | S |  | G | N | S |  | S | R |  | R |
| Hopi Red Flesh^L^ |  | O | M |  | SG | M | S |  | L | B |  | R |
| Jubilee |  | L | L |  | LG | T | W |  | L | R |  | R |
| King & Queen |  | O | M |  | LG | N | W |  | M | B |  | R |
| Legacy |  | L | M |  | LG | N | W |  | M | R |  | R |
| Mickylee |  | R | N |  | LG | N | R |  | M | R |  | S |
| Minilee^L^ |  | R | S |  | G | N | R |  | S | R |  | S |
| Mountain Hoosier |  | O | M |  | SG | T | S |  | L | W |  | R |
| NC Giant^L^ |  | L | G |  | LG | T | R |  | L | R |  | R |
| Navajo Sweet |  | R | S |  | LG | M | W |  | M | R |  | R |
| Peacock WR-60 |  | L | S |  | SG | M | S |  | S | R |  | R |
| Quetzali |  | R | S |  | LG | N | S |  | M | R |  | R |
| Regency F1 |  | O | S |  | MG | M | M |  | S | T |  | R |
| Royal Flush F1 |  | L | M |  | MG | M | N |  | S | B |  | S |
| Sangria F1^L^ |  | O | M |  | MG | M | S |  | S | B |  | S |
| Starbrite F1^L^ |  | O | M |  | LG | L | S |  | S | R |  | R |
| Stars-N-Stripes F1 |  | O | M |  | DG | T | W |  | L | B |  | S |
| Stone Mountain |  | O | M |  | SG | T | S |  | L | T |  | R |
| Sugar Baby |  | R | M |  | MB | S | S |  | S | R |  | S |
| Sugarlee |  | R | S |  | LG | M | W |  | M | R |  | R |
| Sweet Princess |  | O | M |  | G | M | R |  | T | R |  | R |
| Tendersweet OF^L^ |  | E | M |  | DG | M | N |  | L | W |  | O |
| Tom Watson |  | E | M |  | MG | T | S |  | L | T |  | R |
| Yellow Crimson^L^ |  | L | L |  | LG | N | S |  | L | B |  | C |

†Fruit Shape: elongate (E), oval (O), round (R)

‡Fruit Size: micro (<3 lb.) (C), mini (3-8 lb.) (N), icebox (9-13 lb.) (B), small (S), sometimes called pee-wee (14-18 lb.), medium (19-24 lb.) (M), large (25-32 lb.) (L), and giant (>32 lb.) (G).

¶Rind Color: light green (LG), medium green (MG), dark green (DG), solid light black (LB), solid medium black (MB), solid dark black (DB) golden (G), solid green (SG), gray (*R*), Yellow (Y), mottled (M)

§Rind thickness: thick (>10mm) (T), medium (5-10mm) (M), thin (<5mm) (N)

#Rind Pattern: wide stripe (W), medium stripe (M), narrow stripe (N), gray (G), solid (S). Rattle Snake (*R*) [Dark green is dominant, stripe is decided by dark green]

††Seed Size: tomato size (T), small (S), medium (M), large (L)

‡‡Seed Color: black (B), brown (R), tan (T), dotted (D), white (W)

§§Flesh Color: scarlet red (S), coral red (R), orange (O), salmon yellow (Y), canary yellow (C), or white (W)

^L^ Used for lycopene

Table S3. BLUP, regression coefficient (*b_i_*), deviation from regression (*S^2^_d_*), Shukla’s stability variance (*σ_i_^2^*), and Kang’s stability statistics (*YS_i_*) for lycopene of 10 watermelon genotypes tested in 3 year and 8 locations.

|  |  | Lycopene (mg kg^-1^) | | | | | | | | |
| --- | --- | --- | --- | --- | --- | --- | --- | --- | --- | --- |
| Genotype |  | BLUP |  | *b_i_*, |  | *S^2^_d_* |  | *σ_i_^2^* |  | *YS_i_* |
| Allsweet |  | 41.55 |  | 2.132*** |  | 173.723*** |  | 16.28 |  | 7+ |
| Charleston Gray |  | 38.60 |  | 0.678 |  | 42.897* |  | 2.1 |  | 7+ |
| Crimson Sweet |  | 43.83 |  | 1.229 |  | 45.474 |  | 45.50 |  | 10+ |
| Hopi Red Flesh |  | 34.31 |  | 0.589 |  | 154.747*** |  | 57.31 |  | 3 |
| Minilee |  | 52.15 |  | 0.664 |  | 89.554** |  | 42.37 |  | 13+ |
| NC Giant |  | 33.58 |  | 0.762 |  | 100.573*** |  | 42.48 |  | 2 |
| Sangria F1 |  | 48.62 |  | 0.733 |  | 77.692* |  | 26.07 |  | 12+ |
| Starbrite F1 |  | 44.76 |  | 1.003 |  | 108.092*** |  | 51.79 |  | 11+ |
| Tendersweet OF |  | 8.76 |  | 0.412* |  | 4.145 |  | 19.04 |  | -2 |
| Yellow Crimson |  | 8.98 |  | 0.144** |  | 63.139** |  | 52.44 |  | -1 |

*, **, *** Significant different from unity for the regression coefficients or slope (*b_i_* ) and from zero for the deviation from regression (*S^2^_d_*) and Shukla’s stability variance (*σ_i_^2^*) at 0.05, 0.01 and 0.001 levels of probability, respectively.

+ indicate stable according to Kang stability statistics (*YS_i_*).

Table S4. BLUP, regression coefficient (*b_i_*), deviation from regression (*S^2^_d_*), Shukla’s stability variance (*σ_i_^2^*), and Kang’s stability statistics (*YS_i_*) for sugar of 40 watermelon genotypes tested in 3 year and 8 locations.

|  |  | Sugar (°Brix) | | | | | | | | |
| --- | --- | --- | --- | --- | --- | --- | --- | --- | --- | --- |
| Genotype |  | BLUP |  | *b_i_*, |  | *S^2^_d_* |  | *σ_i_^2^* |  | *YS_i_* |
| AU-Jubilant |  | 10.53 |  | 0.42 |  | 0.75 |  | 0.28 |  | 40+ |
| Allsweet |  | 11.69 |  | 0.60 |  | 1.60* |  | 0.09 |  | 18 |
| Big Crimson |  | 10.59 |  | 0.56 |  | 0.87 |  | 0.95 |  | 17 |
| Black Diamond |  | 10.16 |  | 0.17* |  | 1.36 |  | 0.64 |  | 4 |
| Calhoun Gray |  | 10.85 |  | 0.48 |  | 1.85** |  | 1.21 |  | 23+ |
| Calsweet |  | 11.61 |  | 1.09 |  | 2.02 |  | 1.89 |  | 28+ |
| Carolina Cross#183 |  | 8.47 |  | 2.18* |  | 16.18*** |  | 5.92** |  | -10 |
| Charleston Gray |  | 10.82 |  | 0.42* |  | 1.04* |  | 0.35 |  | 25+ |
| Congo |  | 10.45 |  | 2.19** |  | 1.89 |  | 1.23 |  | 11 |
| Crimson Sweet |  | 12.02 |  | 1.04 |  | 0.78 |  | 0.50 |  | 43+ |
| Desert King |  | 10.15 |  | 2.56*** |  | 3.68** |  | 1.77 |  | 5 |
| Early Arizona |  | 10.05 |  | 1.87* |  | 3.44** |  | 1.00 |  | 6 |
| Early Canada |  | 10.51 |  | 0.65 |  | 1.29* |  | 1.33 |  | 10 |
| Fiesta F1 |  | 11.57 |  | 0.59 |  | 1.06 |  | 0.66 |  | 32+ |
| Georgia Rattlesnake |  | 10.59 |  | 2.67*** |  | 3.72** |  | 1.23 |  | 16 |
| Golden Midget |  | 9.64 |  | 0.88 |  | 6.16*** |  | 5.61** |  | -7 |
| Graybelle |  | 11.65 |  | 0.81 |  | 1.24 |  | 0.76 |  | 35+ |
| Hopi Red Flesh |  | 10.51 |  | 0.91 |  | 3.81*** |  | 0.59 |  | 12 |
| Jubilee |  | 10.83 |  | 0.56 |  | 0.72 |  | 0.40 |  | 21+ |
| King & Queen |  | 10.13 |  | 0.82 |  | 1.03 |  | 0.73 |  | 7 |
| Legacy |  | 11.77 |  | 0.48* |  | 0.94* |  | 0.27 |  | 36+ |
| Mickylee |  | 10.89 |  | 0.90 |  | 1.27 |  | 0.37 |  | 22+ |
| Minilee |  | 11.47 |  | 0.49 |  | 1.12* |  | 0.75 |  | 31+ |
| Mountain Hoosier |  | 11.29 |  | 0.95 |  | 1.05 |  | 0.65 |  | 26+ |
| NC Giant |  | 10.42 |  | 1.16 |  | 17.53*** |  | 1.06 |  | 9 |
| Navajo Sweet |  | 9.10 |  | 1.21 |  | 0.95 |  | 9.24** |  | -6 |
| Peacock WR-60 |  | 10.9 |  | 1.27 |  | 6.17** |  | 3.68** |  | 5 |
| Quetzali |  | 11.53 |  | 0.76 |  | 0.91 |  | 0.12 |  | 34+ |
| Regency F1 |  | 11.71 |  | 0.62 |  | 0.80 |  | 0.31 |  | 38+ |
| Royal Flush F1 |  | 11.70 |  | 2.58*** |  | 3.67** |  | 1.19 |  | 42+ |
| Sangria F1 |  | 11.65 |  | 1.53* |  | 1.32** |  | 0.79 |  | 37+ |
| Starbrite F1 |  | 11.58 |  | 0.39* |  | 1.53** |  | 0.38 |  | 41+ |
| Stars-N-Stripes F1 |  | 11.36 |  | 0.69 |  | 1.31 |  | 0.93 |  | 30+ |
| Stone Mountain |  | 9.14 |  | -0.07*** |  | 2.51*** |  | 0.99 |  | 0 |
| Sugar Baby |  | 10.60 |  | 1.12 |  | 0.88 |  | 0.65 |  | 15 |
| Sugarlee |  | 11.57 |  | 0.51 |  | 1.52 |  | 0.52 |  | 33+ |
| Sweet Princess |  | 11.38 |  | 0.86 |  | 0.60 |  | 0.44 |  | 29+ |
| Tendersweet OF |  | 10.79 |  | 0.86 |  | 1.16 |  | 0.17 |  | 14 |
| Tom Watson |  | 9.17 |  | 1.76 |  | 2.70 |  | 1.05 |  | -1 |
| Yellow Crimson |  | 11.19 |  | 0.37 |  | 0.67 |  | 0.81 |  | 24+ |

*, **, *** Significant different from unity for the regression coefficients or slope (*b_i_* ) and from zero for the deviation from regression (*S^2^_d_*) and Shukla’s stability variance (*σ_i_^2^*) at 0.05, 0.01 and 0.001 levels of probability, respectively.

+ indicate stable according to Kang stability statistics (*YS_i_*).

Table S5. BLUP, regression coefficient (*b_i_*), deviation from regression (*S^2^_d_*), Shukla’s stability variance (*σ_i_^2^*), and Kang’s stability statistics (*YS_i_*) for hollowheart resistance of 40 watermelon genotypes tested in 3 year and 8 locations.

|  |  | Hollowheart | | | | | | | | |
| --- | --- | --- | --- | --- | --- | --- | --- | --- | --- | --- |
| Genotype |  | BLUP |  | *b_i_*, |  | *S^2^_d_* |  | *σ_i_^2^* |  | *YS_i_* |
| AU-Jubilant |  | 3.21 |  | 0.69 |  | 0.57* |  | 0.14 |  | 26+ |
| Allsweet |  | 3.24 |  | 0.45 |  | 0.81* |  | 0.15 |  | 20+ |
| Big Crimson |  | 3.56 |  | 2.34 |  | 1.57* |  | 0.68 |  | 38+ |
| Black Diamond |  | 3.42 |  | 1.98 |  | 2.08*** |  | 0.46 |  | 33+ |
| Calhoun Gray |  | 3.20 |  | 0.39 |  | 0.25 |  | 0.10 |  | 15 |
| Calsweet |  | 3.18 |  | -0.29* |  | 0.30 |  | 0.19 |  | 13 |
| Carolina Cross#183 |  | 3.39 |  | 1.33 |  | 1.02 |  | 0.73 |  | 36+ |
| Charleston Gray |  | 3.17 |  | 0.60 |  | 0.62*** |  | 0.49 |  | 32+ |
| Congo |  | 3.41 |  | 1.57 |  | 1.09** |  | 1.18** |  | 29+ |
| Crimson Sweet |  | 3.05 |  | 1.15 |  | 0.43 |  | 0.35 |  | 25+ |
| Desert King |  | 3.41 |  | 0.09 |  | 1.96** |  | 1.18** |  | 27+ |
| Early Arizona |  | 3.28 |  | 2.94* |  | 0.56 |  | 0.18 |  | 23+ |
| Early Canada |  | 3.53 |  | 4.09* |  | 1.11 |  | 0.56 |  | 37+ |
| Fiesta F1 |  | 3.19 |  | 0.50 |  | 0.22* |  | 0.12 |  | 14 |
| Georgia Rattlesnake |  | 3.09 |  | -0.03** |  | 0.19* |  | 0.07 |  | 6 |
| Golden Midget |  | 3.11 |  | 0.09*** |  | 0.78*** |  | 0.18 |  | 7 |
| Graybelle |  | 3.31 |  | 1.23 |  | 0.83 |  | 0.40 |  | 24+ |
| Hopi Red Flesh |  | 3.20 |  | 0.53 |  | 1.05*** |  | 0.45 |  | 19 |
| Jubilee |  | 3.20 |  | 0.91 |  | 0.21 |  | 0.16 |  | 21+ |
| King & Queen |  | 3.12 |  | 0.86 |  | 1.09*** |  | 0.58 |  | 16 |
| Legacy |  | 3.09 |  | 0.07 |  | 0.11 |  | 0.01 |  | 8 |
| Mickylee |  | 3.03 |  | -0.01** |  | 0.08 |  | 0.03 |  | 3 |
| Minilee |  | 3.06 |  | -0.06*** |  | 0.07 |  | -0.01 |  | 2 |
| Mountain Hoosier |  | 3.72 |  | 1.28 |  | 1.91 |  | 1.21 |  | 32+ |
| NC Giant |  | 3.01 |  | 0.56 |  | 0.30*** |  | 0.01 |  | 1 |
| Navajo Sweet |  | 3.11 |  | 0.43 |  | 0.02 |  | 0.27 |  | 11 |
| Peacock WR-60 |  | 3.03 |  | -0.51*** |  | 0.08 |  | 0.02 |  | 0 |
| Quetzali |  | 3.27 |  | 2.62 |  | 0.64 |  | 0.75 |  | 22+ |
| Regency F1 |  | 3.25 |  | 0.99 |  | 0.34 |  | 0.19 |  | 18 |
| Royal Flush F1 |  | 3.16 |  | 0.42 |  | 0.08 |  | 0.03 |  | 9 |
| Sangria F1 |  | 3.18 |  | -0.11* |  | 0.25* |  | 0.14 |  | 12 |
| Starbrite F1 |  | 3.37 |  | 0.91 |  | 0.60 |  | 0.37 |  | 30+ |
| Stars-N-Stripes F1 |  | 3.32 |  | 1.14 |  | 0.31 |  | 0.17 |  | 29+ |
| Stone Mountain |  | 3.12 |  | 0.45 |  | 0.13 |  | 0.04 |  | 4 |
| Sugar Baby |  | 3.13 |  | 1.01 |  | 0.13 |  | 0.06 |  | 4 |
| Sugarlee |  | 3.16 |  | 0.39 |  | 0.16 |  | 0.16 |  | 10 |
| Sweet Princess |  | 3.42 |  | 0.38 |  | 0.73 |  | 0.54 |  | 35+ |
| Tendersweet OF |  | 4.77 |  | 5.14** |  | 6.51*** |  | 1.47 |  | 35+ |
| Tom Watson |  | 3.17 |  | 0.18 |  | 0.27 |  | 0.14 |  | 17 |
| Yellow Crimson |  | 3.70 |  | 2.60 |  | 1.98** |  | 1.32 |  | 33+ |

*, **, *** Significant different from unity for the regression coefficients or slope (*b_i_* ) and from zero for the deviation from regression (*S^2^_d_*) and Shukla’s stability variance (*σ_i_^2^*) at 0.05, 0.01 and 0.001 levels of probability, respectively.

+ indicate stable according to Kang stability statistics (*YS_i_*).


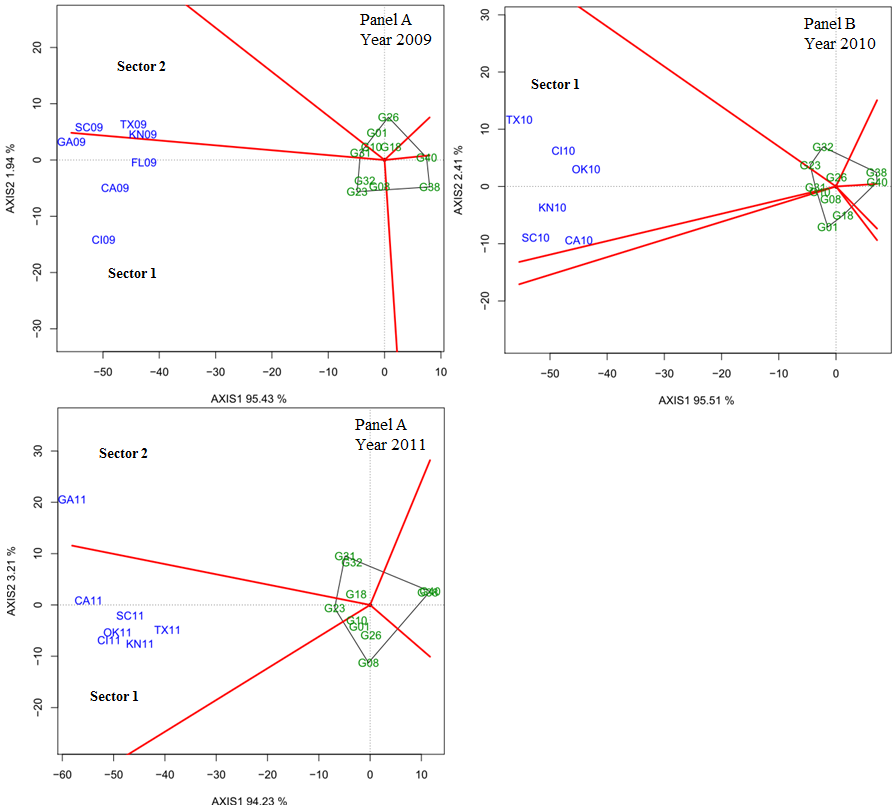


Supplemental Figure 1. The polygon (which–won–where) view of BLUP-genotype main effects plus genotypic x location interaction effect (BLUP-GGL) biplot of 10 watermelon genotypes tested in 3 year (Panel A: 2009, Panel B: 2010, and Panel C: 2011) and 8 locations for lycopene. The biplots were based on Scaling = 0, Centering = 0, and SVP = 2.


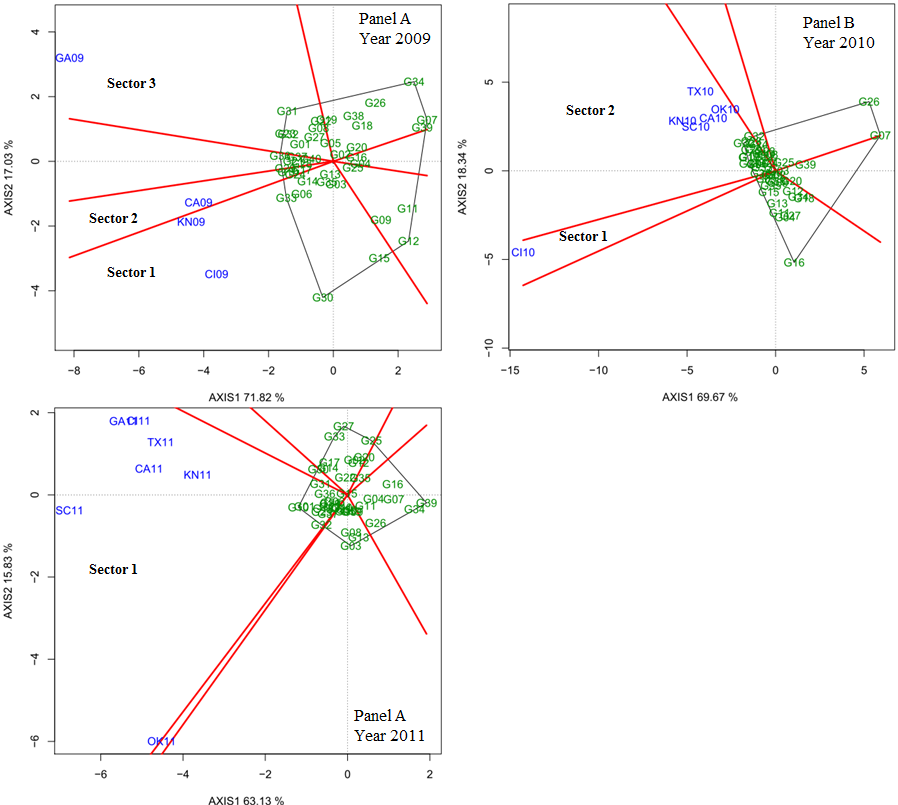


Supplemental Figure 2. The polygon (which–won–where) view of BLUP-genotype main effects plus genotypic x location interaction effect (BLUP-GGL) biplot of 40 watermelon genotypes tested in 3 year (Panel A: 2009, Panel B: 2010, and Panel C: 2011) and 8 locations for sugar. The biplots were based on Scaling = 0, Centering = 0, and SVP = 2.


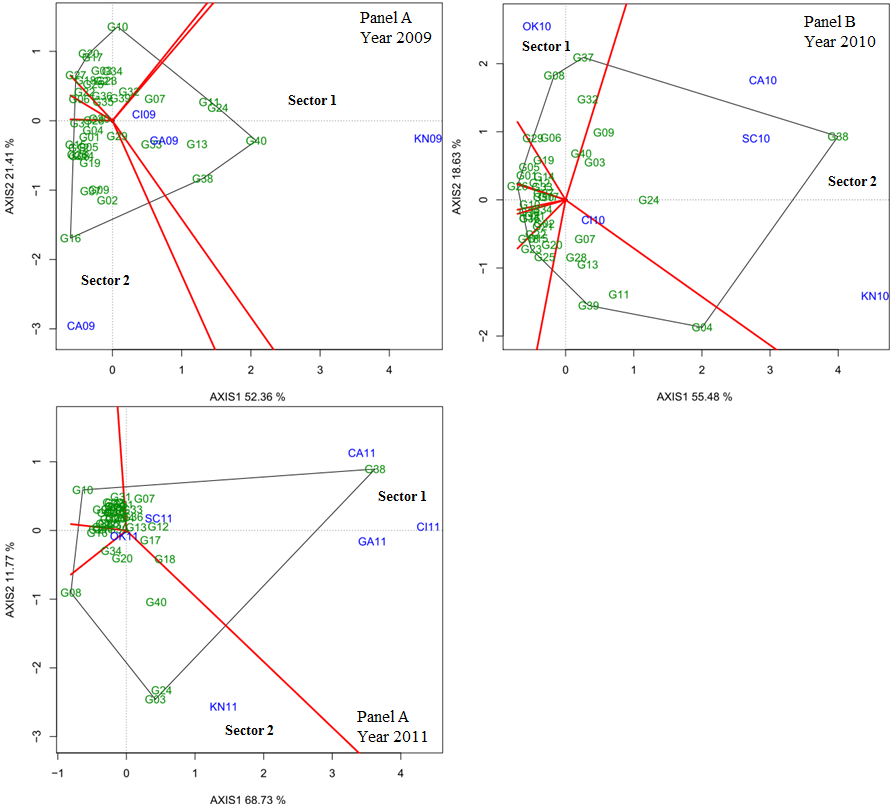


Supplemental Figure 3. The polygon (which–won–where) view of BLUP-genotype main effects plus genotypic x location interaction effect (BLUP-GGL) biplot of 40 watermelon genotypes tested in 3 year (Panel A: 2009, Panel B: 2010, and Panel C: 2011) and 8 locations for hollowheart resistance. The biplots were based on Scaling = 0, Centering = 0, and SVP = 2.


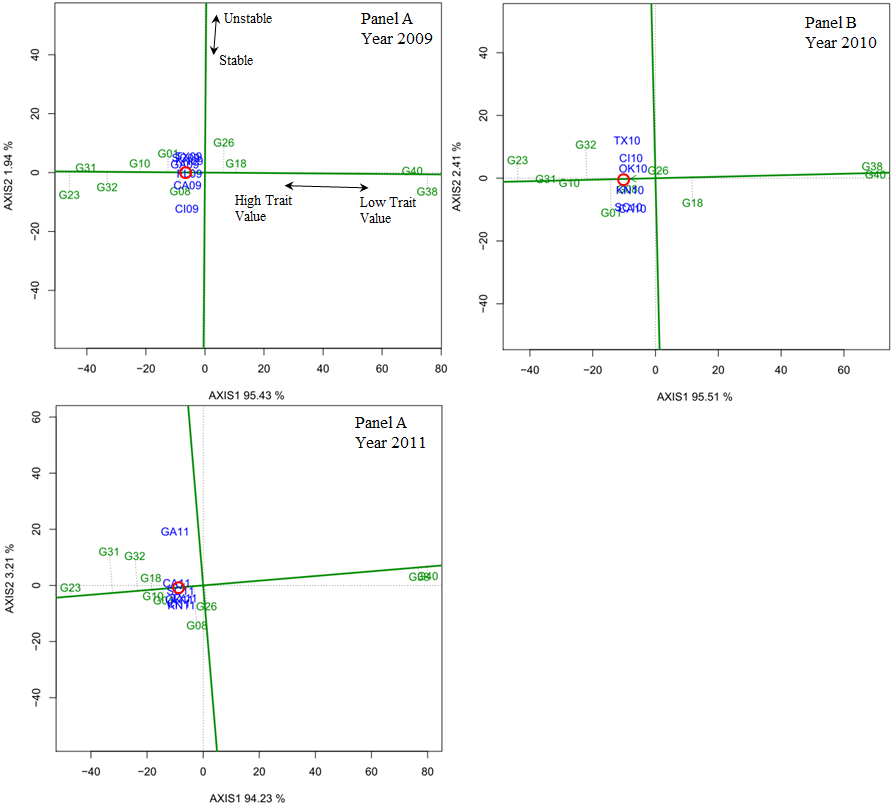


Supplemental Figure 4. The mean vs. stability view of BLUP-genotype main effects plus genotypic x location interaction effect (BLUP-GGL) biplot of 10 watermelon genotypes tested in 3 year (Panel A: 2009, Panel B: 2010, and Panel C: 2011) and 8 locations for lycopene. The biplots were based on Scaling = 0, Centering = 2, and SVP = 1. The ideal genotype is represented by a circle on average environment coordinate (AEC)-abscissa which passed through biplot origin.


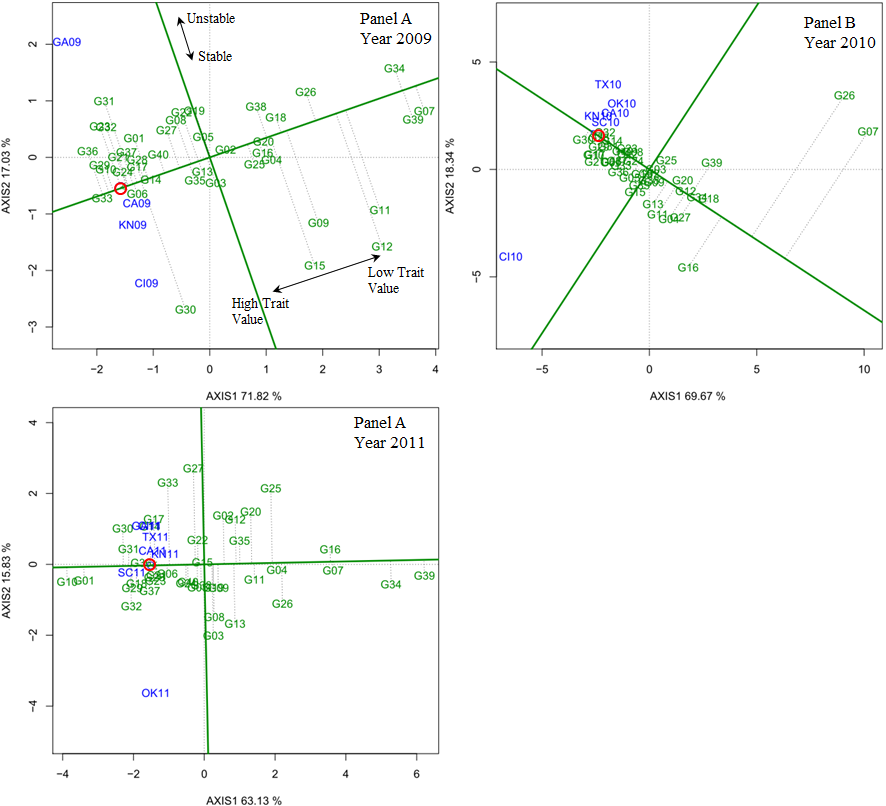


Supplemental Figure 5. The mean vs. stability view of BLUP-genotype main effects plus genotypic x location interaction effect (BLUP-GGL) biplot of 40 watermelon genotypes tested in 3 year (Panel A: 2009, Panel B: 2010, and Panel C: 2011) and 8 locations for sugar. The biplots were based on Scaling = 0, Centering = 2, and SVP = 1. The ideal genotype is represented by a circle on average environment coordinate (AEC)-abscissa which passed through biplot origin.


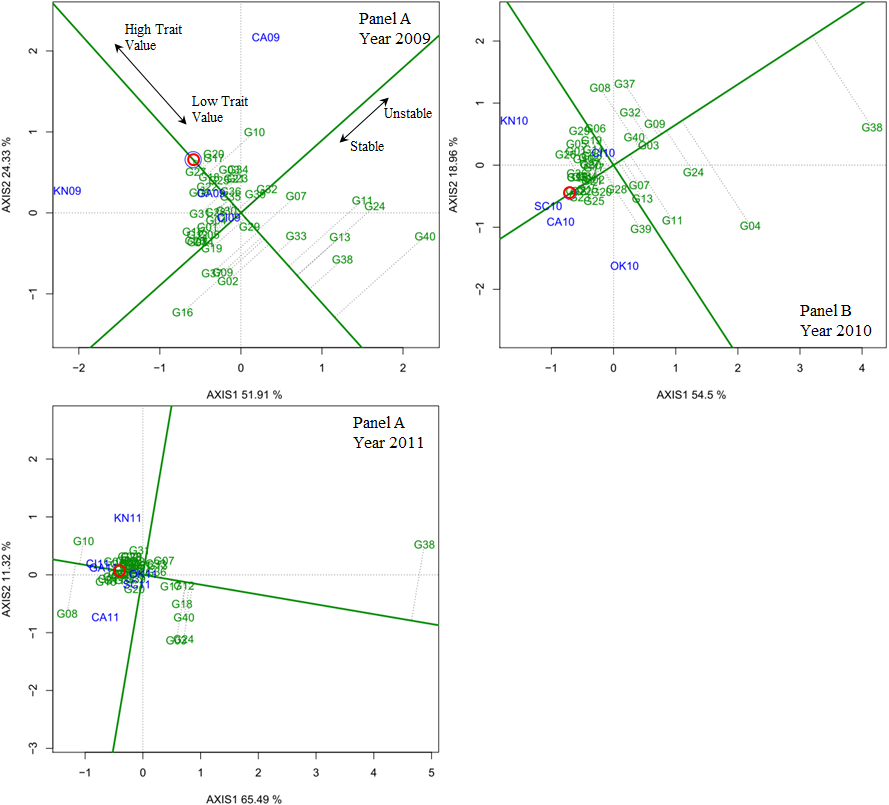


Supplemental Figure 6. The mean vs. stability view of BLUP-genotype main effects plus genotypic x location interaction effect (BLUP-GGL) biplot of 40 watermelon genotypes tested in 3 year (Panel A: 2009, Panel B: 2010, and Panel C: 2011) and 8 locations for hollowheart resistance. The biplots were based on Scaling = 0, Centering = 2, and SVP = 1. The ideal genotype is represented by a circle on average environment coordinate (AEC)-abscissa which passed through biplot origin.


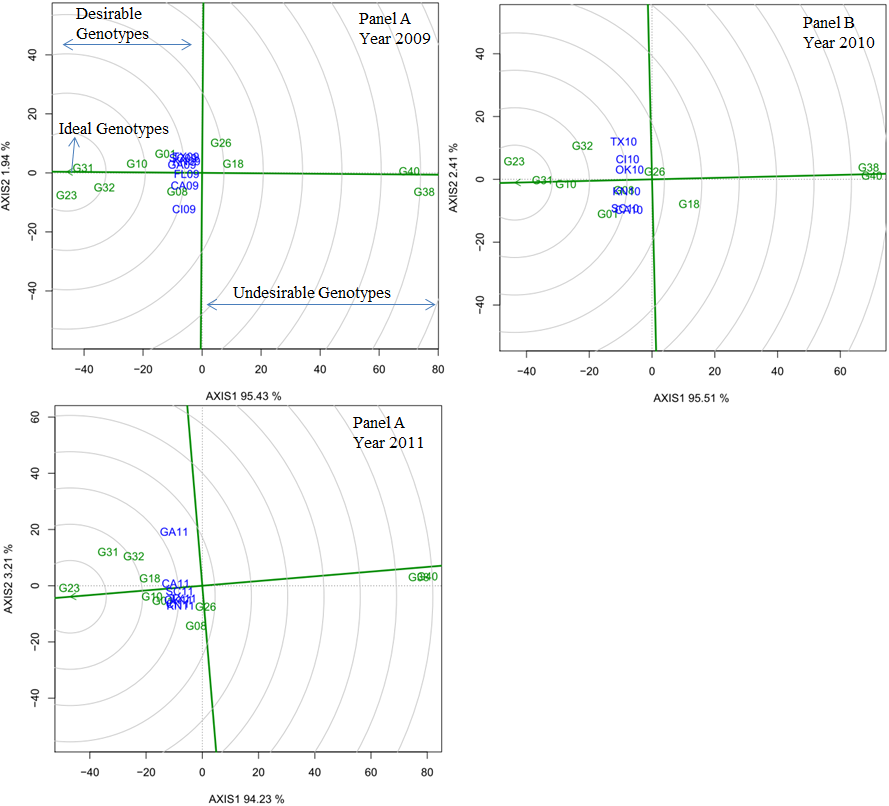


Supplemental Figure 7. The genotypes comparison with ideal genotype view of BLUP-genotype main effects plus genotypic x location interaction effect (BLUP-GGL) biplot of 10 watermelon genotypes tested in 3 year (Panel A: 2009, Panel B: 2010, and Panel C: 2011) and 8 locations for lycopene. The biplots were based on Scaling = 0, Centering = 2, and SVP = 1. An ideal genotype is represented by circle within innermost concentric circles on average environment coordinate (AEC)-abscissa which passed through biplot origin.


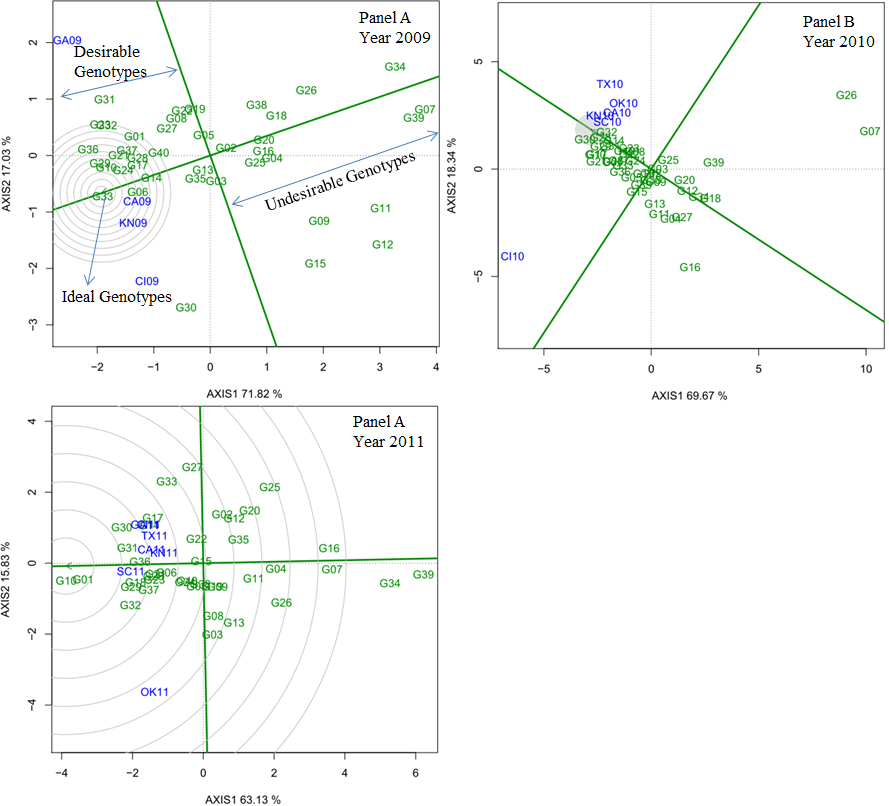


Supplemental Figure 8. The genotypes comparison with ideal genotype view of BLUP-genotype main effects plus genotypic x location interaction effect (BLUP-GGL) biplot of 40 watermelon genotypes tested in 3 year (Panel A: 2009, Panel B: 2010, and Panel C: 2011) and 8 locations for sugar. The biplots were based on Scaling = 0, Centering = 2, and SVP = 1. An ideal genotype is represented by circle within innermost concentric circles on average environment coordinate (AEC)-abscissa which passed through biplot origin.


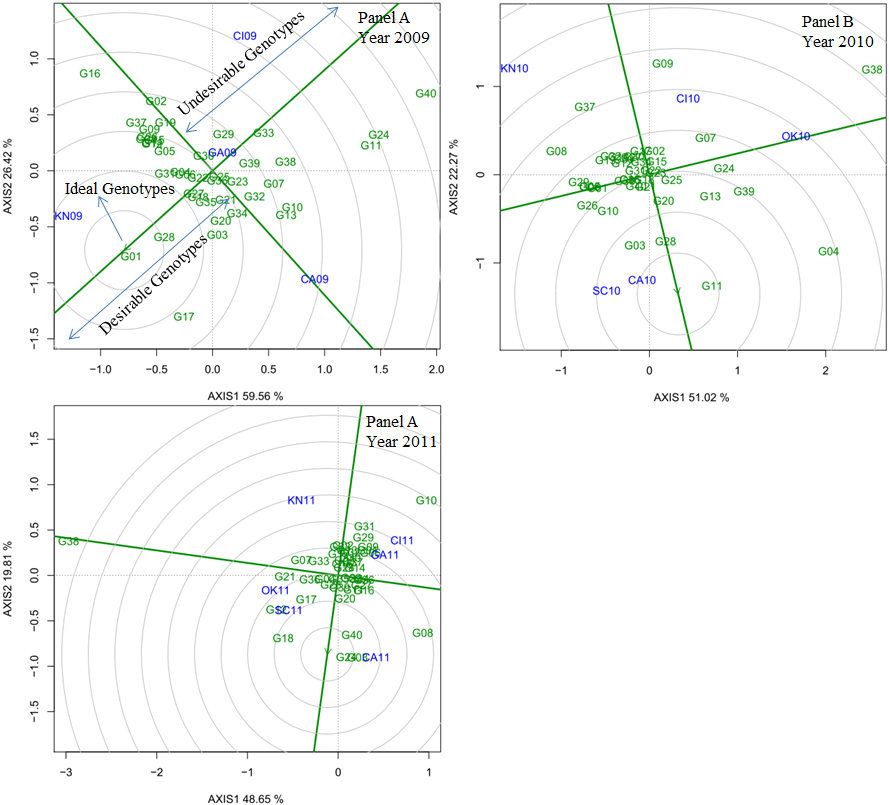


Supplemental Figure 9. The genotypes comparison with ideal genotype view of BLUP-genotype main effects plus genotypic x location interaction effect (BLUP-GGL) biplot of 40 watermelon genotypes tested in 3 year (Panel A: 2009, Panel B: 2010, and Panel C: 2011) and 8 locations for hollowheart resistance. The biplots were based on Scaling = 0, Centering = 2, and SVP = 1. An ideal genotype is represented by circle within innermost concentric circles on average environment coordinate (AEC)-abscissa which passed through biplot origin.
