# Supplementary figures and images for: Stability of fruit quality traits in diverse watermelon cultivars tested in multiple environments
Source: Hortic Res. 2016 Dec 21;3:16066–. doi: 10.1038/hortres.2016.66 (PMC5174159; doi:10.1038/hortres.2016.66)

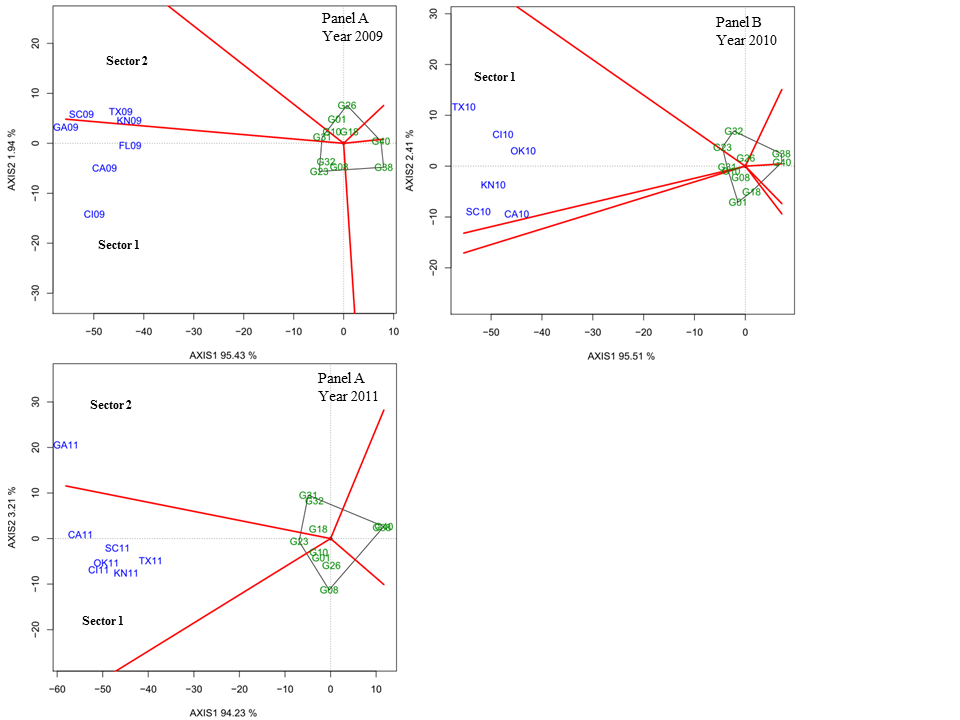

Supplement: Supplementary Figure 1 [file hortres201666-s2.tiff]

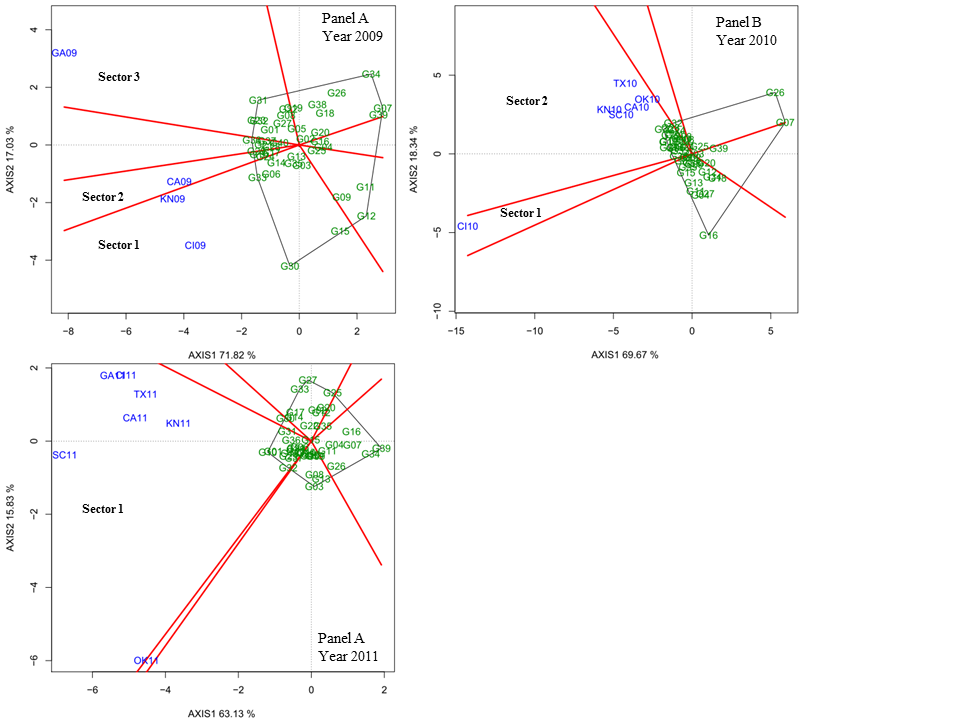

Supplement: Supplementary Figure 2 [file hortres201666-s3.tiff]

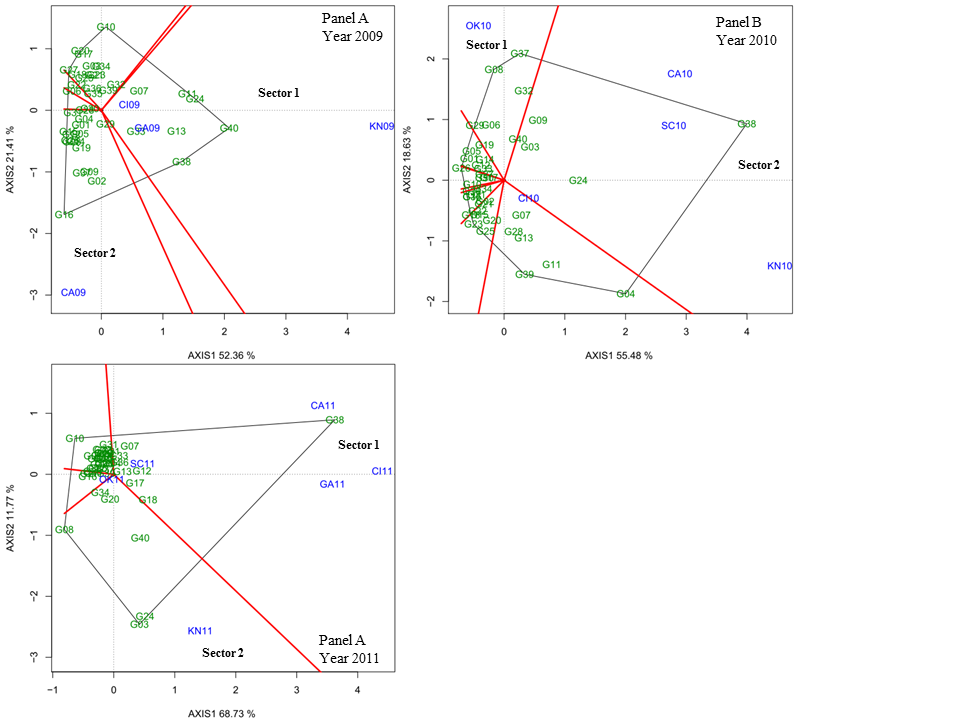

Supplement: Supplementary Figure 3 [file hortres201666-s4.tiff]

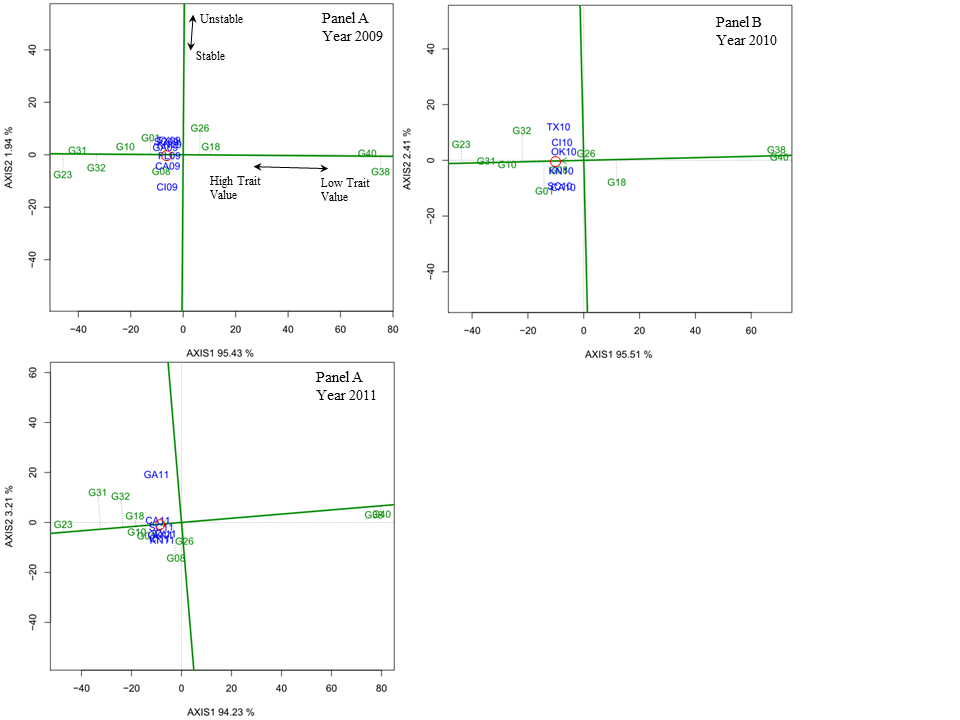

Supplement: Supplementary Figure 4 [file hortres201666-s5.tiff]

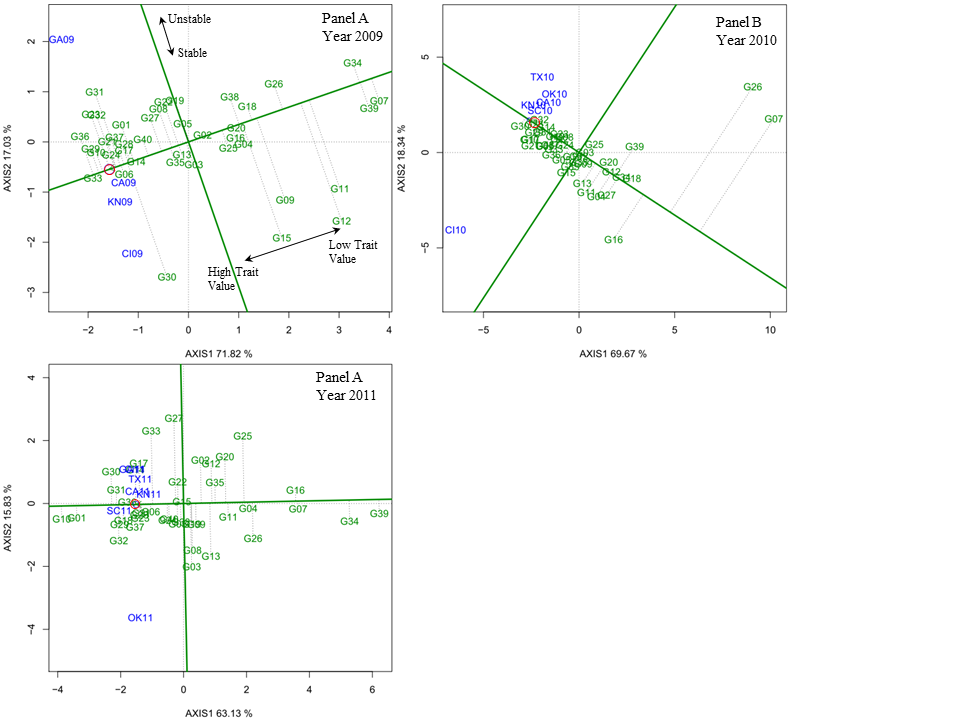

Supplement: Supplementary Figure 5 [file hortres201666-s6.tiff]

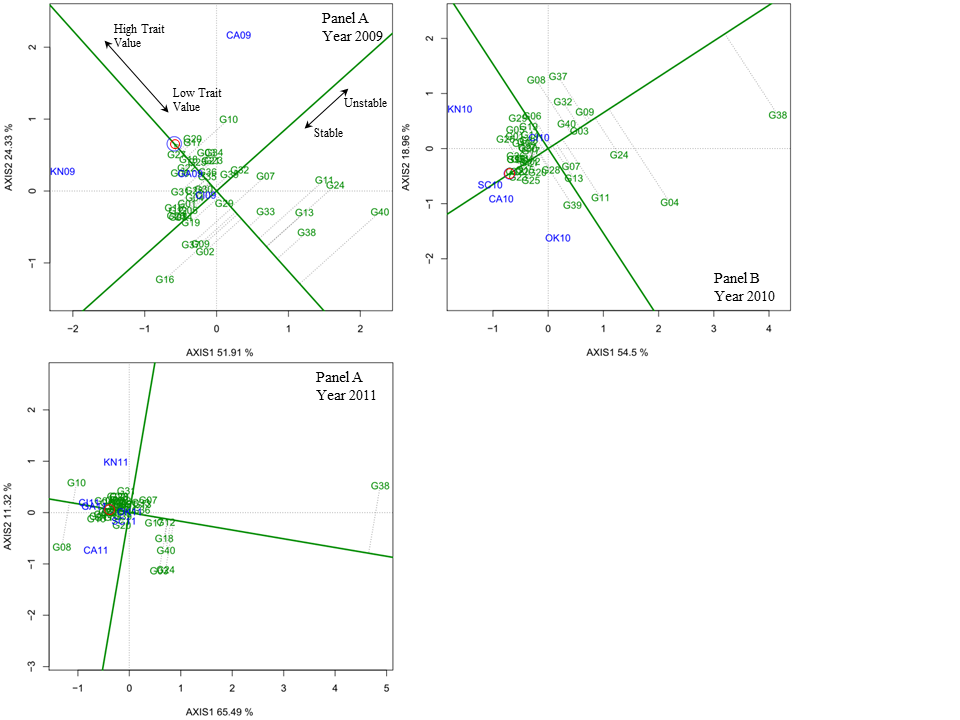

Supplement: Supplementary Figure 6 [file hortres201666-s7.tiff]

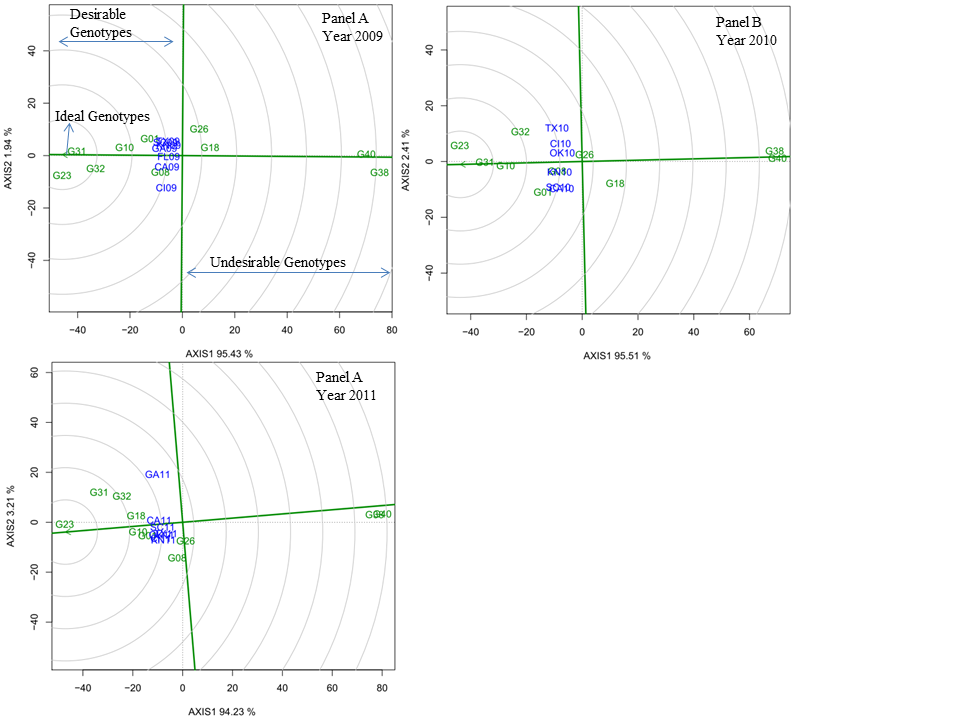

Supplement: Supplementary Figure 7 [file hortres201666-s8.tiff]

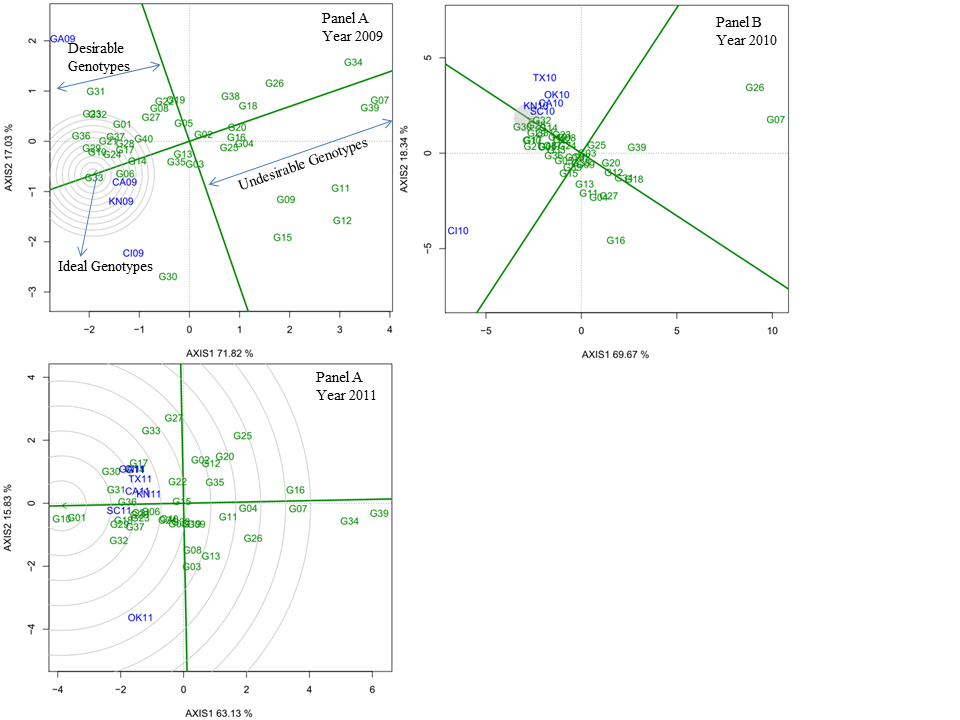

Supplement: Supplementary Figure 8 [file hortres201666-s9.tiff]

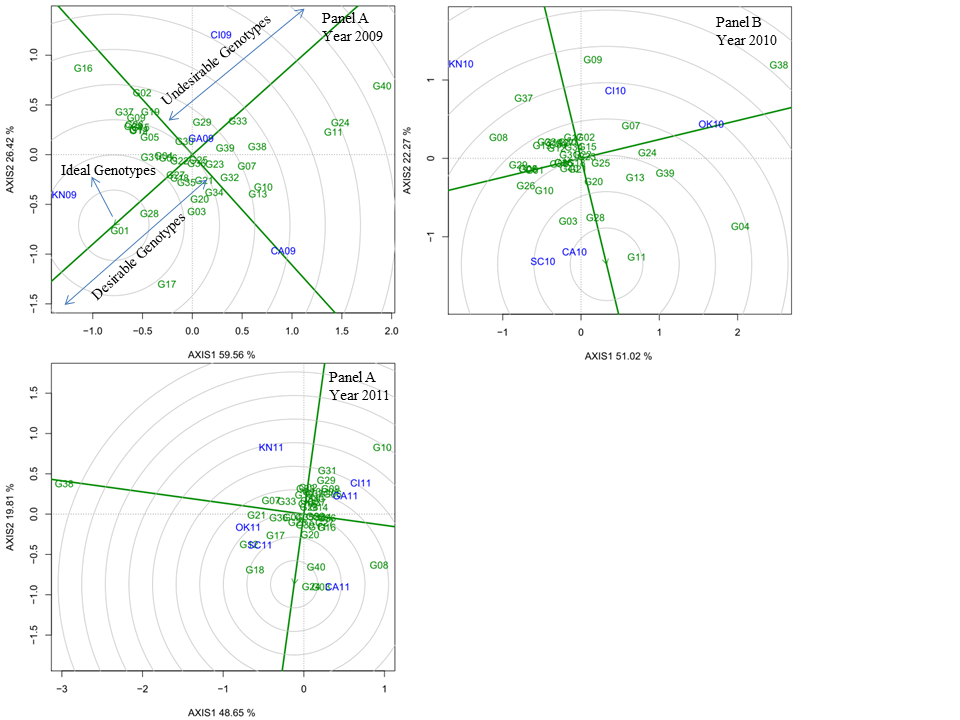

Supplement: Supplementary Figure 9 [file hortres201666-s10.tiff]
